# Automated detection of elephants in wildlife video

**DOI:** 10.1186/1687-5281-2013-46

**Published:** 2013-08-01

**Authors:** Matthias Zeppelzauer

**Affiliations:** Interactive Media Systems Group, Institute for Software Technology and Interactive Systems, Vienna University of Technology, Favoritenstrasse 9-11, Vienna 1040, Austria

**Keywords:** Automated video indexing, Color-based object detection, Automatic animal detection, Elephant detection, Object tracking

## Abstract

Biologists often have to investigate large amounts of video in behavioral studies of animals. These videos are usually not sufficiently indexed which makes the finding of objects of interest a time-consuming task. We propose a fully automated method for the detection and tracking of elephants in wildlife video which has been collected by biologists in the field. The method dynamically learns a color model of elephants from a few training images. Based on the color model, we localize elephants in video sequences with different backgrounds and lighting conditions. We exploit temporal clues from the video to improve the robustness of the approach and to obtain spatial and temporal consistent detections. The proposed method detects elephants (and groups of elephants) of different sizes and poses performing different activities. The method is robust to occlusions (e.g., by vegetation) and correctly handles camera motion and different lighting conditions. Experiments show that both near- and far-distant elephants can be detected and tracked reliably. The proposed method enables biologists efficient and direct access to their video collections which facilitates further behavioral and ecological studies. The method does not make hard constraints on the species of elephants themselves and is thus easily adaptable to other animal species.

## 1 Introduction

Many biologists study the behavior of free-ranging animals in the field. For this purpose they collect large video corpora which include monitoring video, videos from field trips, and personally recorded wildlife video footage [1]. The result of this data collection is the large amounts of video which sometimes span several hundreds of hours. Unfortunately, the access to the videos is limited because objects (e.g., the presence of a particular animal) and events of interest (e.g., particular animal behaviors) are not indexed. In many cases only (handwritten) field notes exist from the recording sessions. For manual indexing biologists have to browse linearly through the videos to find and describe objects and events of interest. This is a time-consuming and tedious task for large amounts of videos [2]. Since indexing should preferably be performed by domain experts, it quickly becomes an expensive task. Visual analysis methods have the ability to significantly accelerate the process of video indexing and enable novel ways to efficiently access and search large video collections.

Wildlife recordings captured in the field represent a challenging real-life scenario for automated visual analysis. While a lot of research has been performed on the visual analysis of human beings and human-related events, the automated analysis of animals has been widely neglected in the past. Existing approaches on animal analysis frequently operate in highly controlled environments, for example, with a fixed camera, in a well-defined location, with static background, and without interfering environmental factors, such as occlusions, different lighting conditions, and interfering objects [3,4]. A typical example for a controlled setting is the monitoring applications where the camera is usually fixed and the background is mostly static [5]. In such a scenario we can easily learn a background model and identify objects of interest by detecting changes to the background. The video material we investigate in this work does not provide such a well-defined setting.

We are provided with a large collection of wildlife videos captured by biologists in the field. The videos have been captured during different field trips and serve as a basis for the investigation of the behavior and communication of African elephants. The videos show a large number of different locations, elephants and elephant groups of different sizes, poses, and distances to the camera. In many sequences elephants are partly or completely absent. Assumptions and constraints of specialized approaches (derived from controlled environments) do not hold for such unconstrained video footage. The question arises as to which degree visual analysis methods can facilitate the access to such video collections.

**Figure 1:**
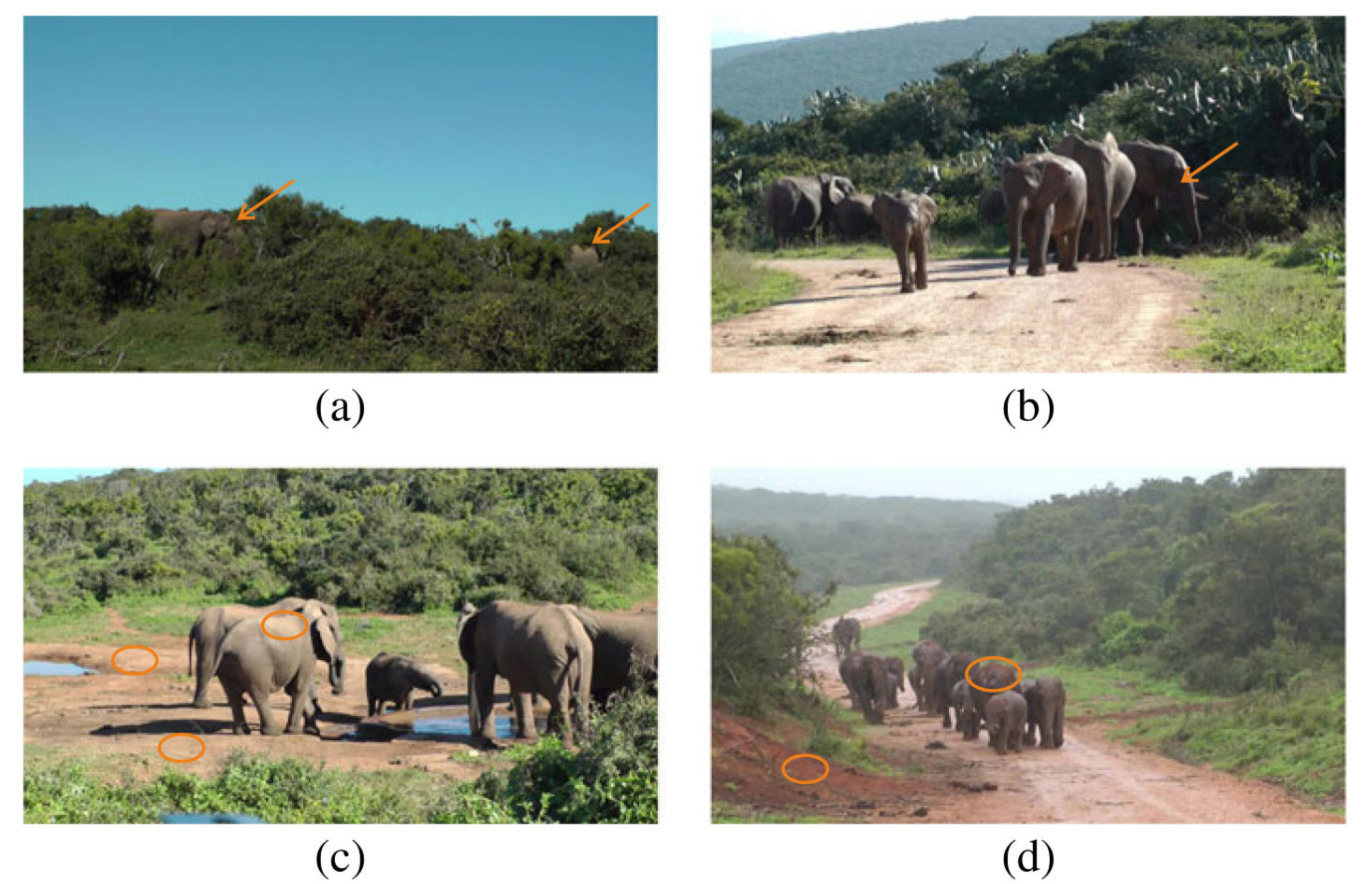
Challenges of detecting elephants in their natural habitat **(a)** Two highly occluded elephants. This example shows that shape is not a good indicator for elephant detection. **(b)** A group of elephants. Due to bad lighting conditions, the elephant labeled with an arrow has a very low contrast and is thus difficult to detect, especially in front of the background (plants) which is dark as well. **(c)** Elephants at a water hole. The encircled areas show regions on the ground and on the elephant’s skin which have similar color and are thus difficult to distinguish. **(d)** Elephants on a wet trail. The elephant’s skin is partly covered by mud from the ground.

Animals are among the most difficult objects for classification and recognition [6]. The detection of elephants is especially hard because their skin does not exhibit a salient texture pattern (like for example, the skin of zebras) and thus lacks in distinctive visual features [7]. Figure 1 shows some images from our video collection that illustrate the typical factors that impede automated detection. Elephants are often occluded by plants and trees, and thus only the body parts are visible. Additionally, elephants are visible in different poses and sizes and in groups or as individuals. The contrast is partly low due to bad lighting conditions, and the elephant skin covers a broad spectrum of colors and shades and is often difficult to separate from the background (e.g., sandy and earthy ground).

We develop a method for the automated detection and tracking of elephants in wildlife video. The method does not make any assumptions about the environment and the recording setting. In a first step we learn a color model of the elephants from a small set of annotated training images. Learning the model does not include domain knowledge and explicitly specified constraints about elephants and their environment. The trained model is applied to individual frames of wildlife video sequences to identify candidate detections. Next, we track the candidate detections over time and join temporally coherent detections in consecutive frames. As a result we obtain spatiotemporally consistent detections which provide additional (stronger) clues for the detection of elephants. At the same time we obtain all information necessary to track the elephants in space and time.

Experiments show that the proposed method yields high performance on wildlife video. We are able to detect and track elephants of different sizes, poses, and distances to the camera. The method is robust to occlusions, camera motion, different backgrounds, and lighting conditions. Most elephants can be detected and tracked successfully (above 90%), while the number of false detections is small (below 5%).

The paper is organized as follows. In Section 2 we survey the related work on the automated visual analysis of animals. Section 3 describes the proposed method for elephant detection, and Section 4 presents the employed wildlife video collection and the experimental setup for our evaluation. We show qualitative and quantitative results of elephant detection in Section 5. Finally, we draw conclusions and summarize our main findings in Section 6.

## 2 Related work

The analysis of animals and animal behavior that is a complex task for computer vision has been rarely addressed so far [6]. Recently, methods related to the analysis of animals have been introduced for different tasks such as species classification [8], gait recognition [9], individual animal recognition [10], and the detection of animal-related events [11]. The basis for most tasks is the *detection* of animals in an image or video stream. In the following discussion, we provide an overview of the different approaches for the detection of animals whereby we follow a path from highly restricted approaches (e.g., semiautomatic approaches) to less-constrained methods (e.g., methods building upon unsupervised learning).

Many approaches on automated animal analysis require human interaction for the detection of animals. For example in [12] the authors present a method for the identification of salamanders by dorsal skin patterns. The method requires that key points along the skeleton of the animal are labeled manually by the user. Similar user input is required in [10] for the identification of elephants from their ear profile. Authors in [13,14] rely on user-defined regions of interest as a basis for the identification of animals.

Other approaches restrict the recording setting or the video material to reduce the complexity of animal detection. Authors in [3] classifies animals using a highly constrained setup with a static camera mounted at one side of a corridor. This setup makes the detection of animals passing the corridor trivial. Alternatively, some methods require that animals take a specific pose towards the camera and then apply, for example, face detection [15] or the detection of other characteristic body parts [16].

A popular clue for the detection of animals is motion. Methods that exploit motion often set hard constraints on the recording setting and the environment. In [5] the underlying assumption is that the background is static and can easily be subtracted. All blobs that remain after background subtraction are treated as candidate detections. While this works well in restricted domains, e.g., for underwater video [5], such assumptions do not hold in more general settings. A method applicable to moving backgrounds (e.g., due to camera motion) is presented in [9] and [17]. The authors track sparse feature points over time and apply RANSAC to separate foreground and background motion. Thereby, the background motion is assumed to be the dominant motion in the scene. The remaining motion is assumed to belong to a single object which is the animal of interest. Other moving objects would disturb the approach and may be falsely detected as animals. Authors in [4] propose a method for animal species detection and make similar constraints concerning the foreground objects in the video: While the camera is static in the investigated setting, the detector requires that the foreground objects are in motion. If several moving foreground objects are detected, the one with the largest motion component is considered to be the animal of interest and all other objects are rejected. This assumption is highly specific to the particular setting and not valid in the context of wildlife video where several animals may be present at the same time.

In real-life settings with unconstrained video material, the detection of animals by specialized detectors becomes unsuitable and does not work reliably due to the large number of unpredictable environmental influences, like occlusions, lighting variations, and background motion. Only a limited number of approaches has been introduced that faces the challenges of unconstrained wildlife video. A method for the detection and tracking of animals in wildlife video is proposed by Burghardt and Ćalić [15]. The authors apply the face detector by Viola and Jones [18] trained for a particular animal species. Once an animal face is detected, the authors try to track it over time. Similar to our work, a tracking scheme is proposed that allows gaps in tracking. Gaps in the context of [15] occur, for example, when an animal turns its head away from the camera. The approach can be applied to different animal species by using adequately trained detectors. However, the face detector of Viola and Jones requires a large training set to learn the dominant face characteristics of a given species. For the detection of lion faces in [15], a training set of 680 positive and 1,000 negative images is employed. Our approach requires only a minimal training set of 10 to 20 images. This significantly reduces the efforts of building a training set, makes the approach more convenient for the actual users (e.g., biologists), and increases the applicability of our approach to new video footage. An advantage of using a well-trained face detector is that the confidence of the resulting detections is relatively high since faces represent particularly distinctive patterns. However, face detection requires the animals to look into the direction of the camera which is, in general, not given in wildlife video.

The authors of [11] present a method for the detection of hunt scenes in wildlife footage. Since hunt scenes are characterized by a significant amount of motion, detection relies on the classification of moving regions. First, color and texture features are extracted for each pixel. Next, each pixel is classified by an artificial neural network to either belong to the animal class or not. A moving region is classified as an animal if the majority of pixels in the region are assigned to the animal class. Operating on individual pixels is computationally expensive and introduces noise. We apply image segmentation as a preprocessing step and perform detection at the segment level. The segmentation improves the robustness of detection and obviates the need for a postprocessing of noisy pixel-based detections. Furthermore, our approach does not rely on motion clues, which additionally enables the detection of animals which are resting or moving slowly.

An interesting approach for the detection and tracking of animals is proposed in [7]. The authors build models of animals in an unsupervised manner from candidate segments detected consistently over successive frames. The candidate segments are obtained from a rectangle detector which uses Haar-like templates at different scales and orientations. For each segment a feature vector is constructed which consists of a color histogram and the rectangle’s width and height. The authors cluster the segments and identify temporally consistent and visually similar rectangles within each cluster. For the detection of animals, the authors extract a texture descriptor from the temporally consistent segments based on SIFT and match it against a precomputed library of animal textures. The authors of [7] report satisfactory results for animals with textured skin, such as zebras, tigers, and giraffes. The authors state that the detection of animals such as elephants and rhinoceroses is hard because their hides are homogeneous and they do not exhibit a distinct texture. The authors further state that their approach is only applicable to videos with single animals and with little background clutter. Both conditions are not met in wildlife video.

We observe an explicit trend towards highly textured animals from computer vision literature which focuses on animals. The ‘favorite’ species are apparently zebras, giraffes, and tigers; see for example [7,9,13,14]. One reason for this bias is that animals with a distinct texture are easier to discriminate from the background. The visual detection of animals without a distinctive texture is hard because only weak visual clues, such as color, exist that can be exploited for detection (a more detailed discussion of visual clues is provided in the following section).

There is rarely work on the visual analysis of species with poorly textured skin such as elephants. The species of elephants is addressed only marginally, e.g., for image classification in [8]. To our knowledge no work on the automated visual detection of elephants in wildlife video has been performed so far. In this article we present a novel approach for the detection of elephants in their natural habitat. The approach enables a more efficient access to wildlife video collections and thus bears the potential to support biologists in behavioral studies.

## 3 Methodology

Knowledge about the environment and the recording setup is an important factor for designing automated visual detectors because it enables the derivation of constraints and visual clues that facilitate detection. In an uncontrolled environment like wildlife video, as investigated in this work, the identification of robust constraints and clues is difficult. The video material we investigate has been captured by different people with a hand camera. Recordings were partly made in an *ad hoc* fashion. This means that we cannot make assumptions about the environment and the camera operation. As a consequence, we have to rely on the very basic visual cues such as shape, texture, motion, and color for the detection of elephants. Prior to the design of our method, we have investigated the suitability of the different visual cues.

A straightforward clue for the detection of elephants is their *shape*. Elephants have a characteristic shape, especially due to their trunk. In practice however shape is not applicable for the detection of elephants in the field because elephants in different poses and viewed from different directions may have diverse shapes. Additionally in most cases, parts of the animals are occluded and only certain body parts are visible which results in arbitrary shapes, as shown in the introductory examples in Figure 1. Similar conclusions are also drawn in [11] for animal detection.

*Texture* may be another useful clue, since elephant skin has numerous fine wrinkles. However, the resulting texture has such a fine granularity that it is not detectable in practice from a reasonable distance to the camera. While texture is not directly applicable to the detection of elephants, we show in Section 3.5 how texture information can be exploited to make the detection of elephants more robust.

*Motion* is another important visual clue for automated detectors [11]. Even if we compensate for camera motion, the remaining object motion of elephants provides only weak clues since elephants move slowly and often remain stationary for a long time. This is especially a problem when the animals are far away from the camera. In such cases motion can hardly be exploited.

A more promising visual clue is *color*. The skin color of elephants covers different shades of brown and gray. Additionally, the skin color is highly influenced by lighting (highlights and backlight) resulting in shades of very light and dark gray, respectively. However, color represents only a weak and ambiguous clue since many objects in the environment (e.g., different grounds and rocks) have similar colors to elephants and easily provoke false-positive detections. In our investigations we observe that color is well-suited as an initial visual clue for the detection of elephants. However, additional clues are necessary to make the detection more robust.

Since we work with video, *temporal clues* are another important source of information. Elephants do not appear and vanish abruptly in the course of time. We exploit temporal relationships between detections in subsequent frames to improve the robustness of detection.

**Figure 2:**
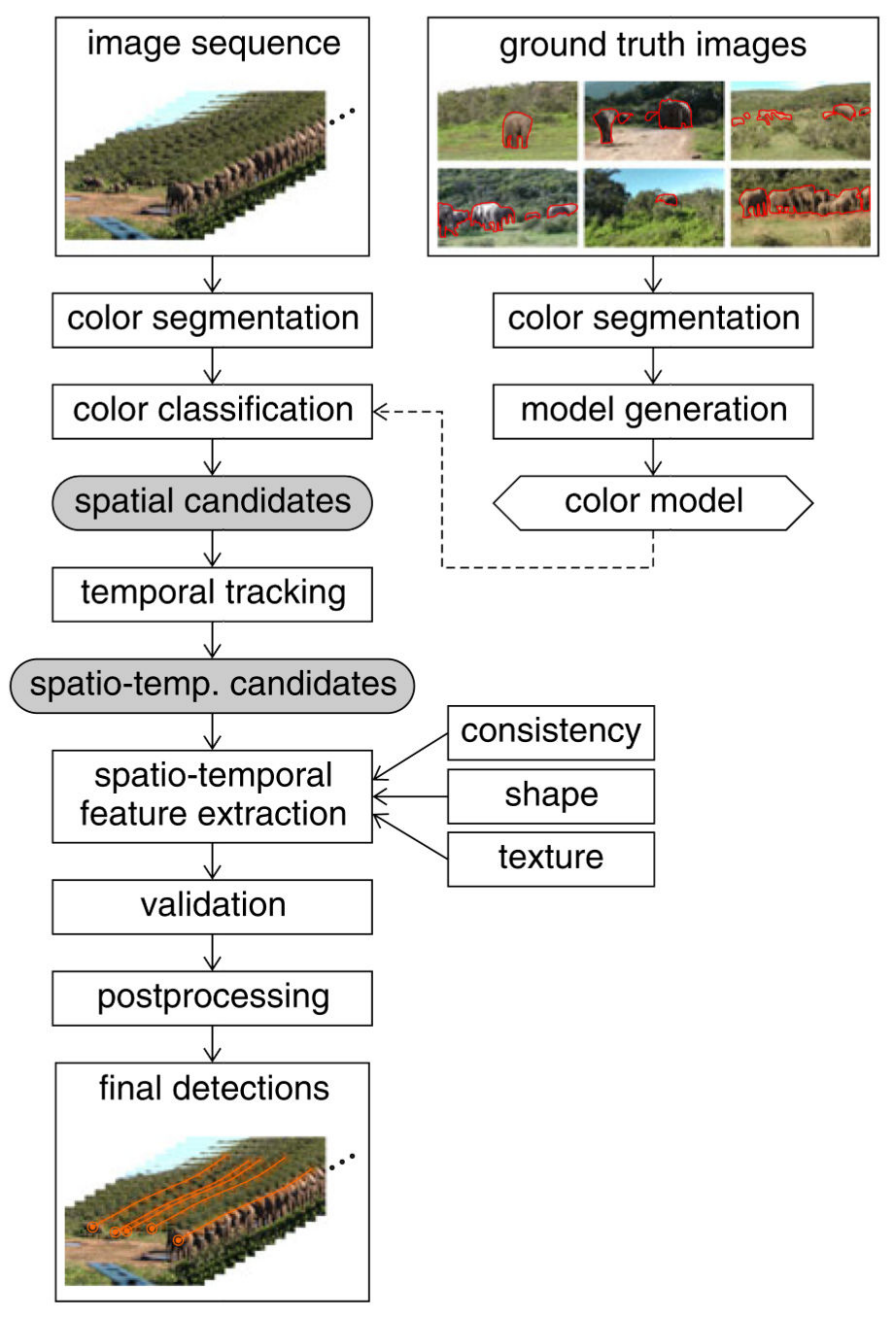
Overview of automated elephant detection First, a color model is generated from labeled ground truth images. Next, image segments are classified by the color model. Positively detected segments (candidates) are tracked through the sequence resulting in spatiotemporally coherent candidates. The final detections are obtained by validating the spatiotemporal candidates by shape, texture, and consistency constraints. Finally, postprocessing fills gaps in tracking for each detection.

An outline of the proposed approach is shown in Figure 2. In a preprocessing step we perform color segmentation of the input images to reduce the amount of data to process and to obtain a higher abstraction level for the following analyses. From manually labeled ground-truth images, we first learn a color model of the elephants. The color model is applied to an input image sequence and detects segments which potentially represent elephants. Next, we track the positively detected segments (candidates) across the image sequence and join them into independent sets of spatiotemporally coherent candidates. From these candidates we extract spatiotemporal features to validate the detections. Detections that pass validation are input to postprocessing where tracking errors are corrected.

### 3.1 Preprocessing

The goal of preprocessing is to reduce the amount of data for processing and to obtain a more abstract representation of the input image sequence. We first downscale the input images (full HD resolution) by a factor of 0.25 to speed up subsequent operations. Next, we perform color segmentation of the images by mean-shift clustering [19]. Prior to segmentation we transform the images to the LUV color space. The LUV color space is a perceptually uniform space. It better approximates color similarity perception than the RGB space and allows similarity judgments using Euclidean distance [20]. After segmentation for each segment, the mean color of all covered pixels is computed and stored as a representative color for each segment.

**Figure 3:**
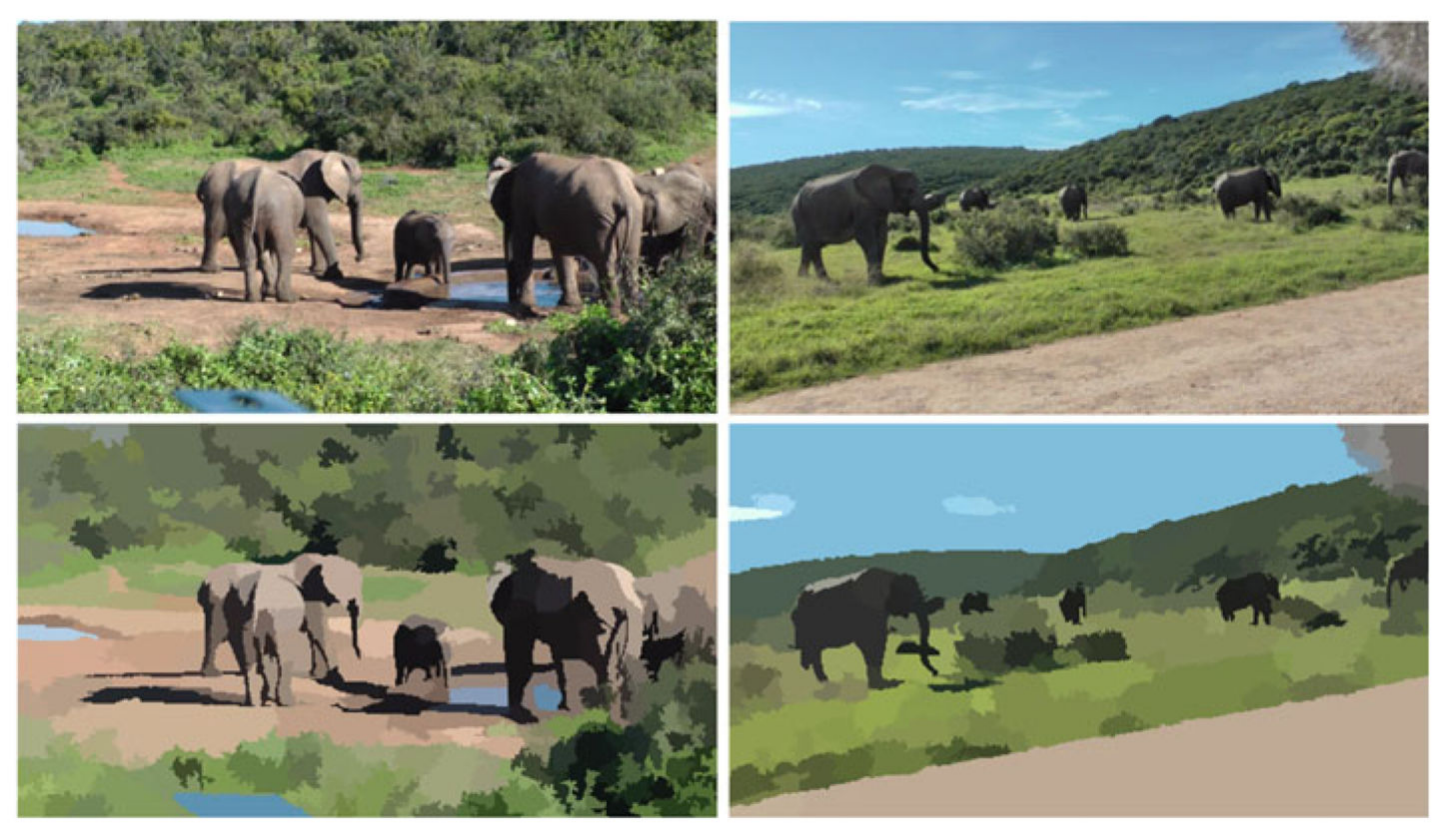
Results of image segmentation for two example images Top: original images, bottom: color segmentation. The color segmentation abstracts from fine details in the images while it preserves object boundaries well.

Color segmentation yields a more abstract representation of the input images in terms of coherent color segments. Additionally, segments usually represent adjacent pixels that belong to the same object. Thus, the representation at the segment level is more expressive than the original representation at the pixel level. Figure 3 shows results of color segmentation for two example images. All subsequent processing steps are performed on the extracted color segments rather than on individual pixels.

### 3.2 Model generation

We learn a discriminative color model of elephant skin from a small set of labeled training images. The model represents foreground colors representing elephants as well as background colors from the surrounding environment. The training images represent different environments and differently shaded elephants in varying lighting situations.

**Figure 4:**
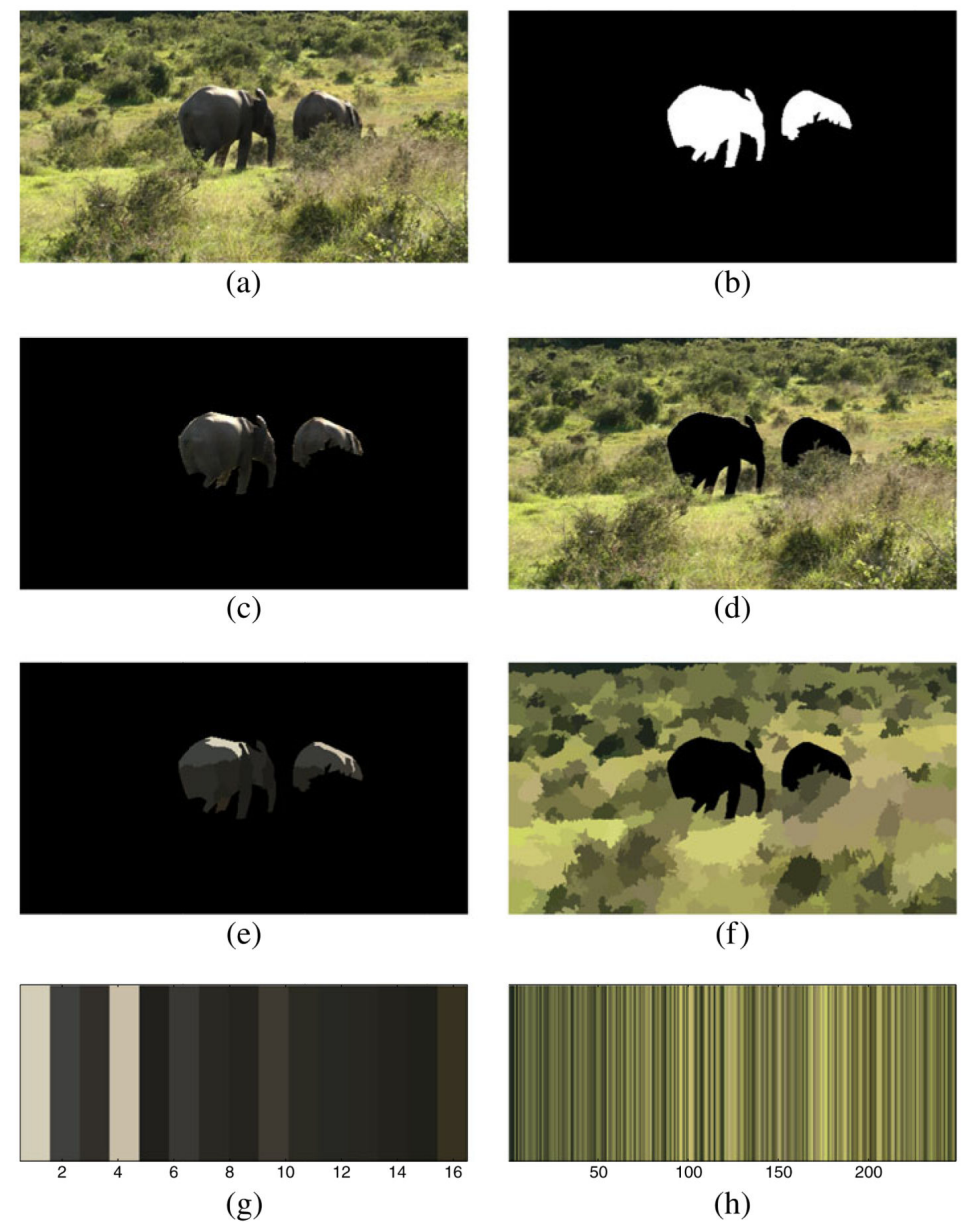
Color model generation from labeled training images **(a)** The input image. **(b)** The manually created labels. **(c)** The foreground image. **(d)** The background image. **(e,f)** The color segmentations of foreground and background image. **(g,h)** The colors of the foreground and background segments.

We first manually label all elephants in the training images (see Figure 4a,b for an example image and the corresponding labeling). Next we split the images into foreground and background based on the manual labeling (see Figure 4c,d). Both the foreground image and the background image are preprocessed and segmented as described in Section 3.1 (see Figure 4e,f). For each foreground and background segment, we take the mean color and transform it to the LUV color space. Figure 4g,h shows the respective colors before conversion to the LUV space.

**Figure 5:**
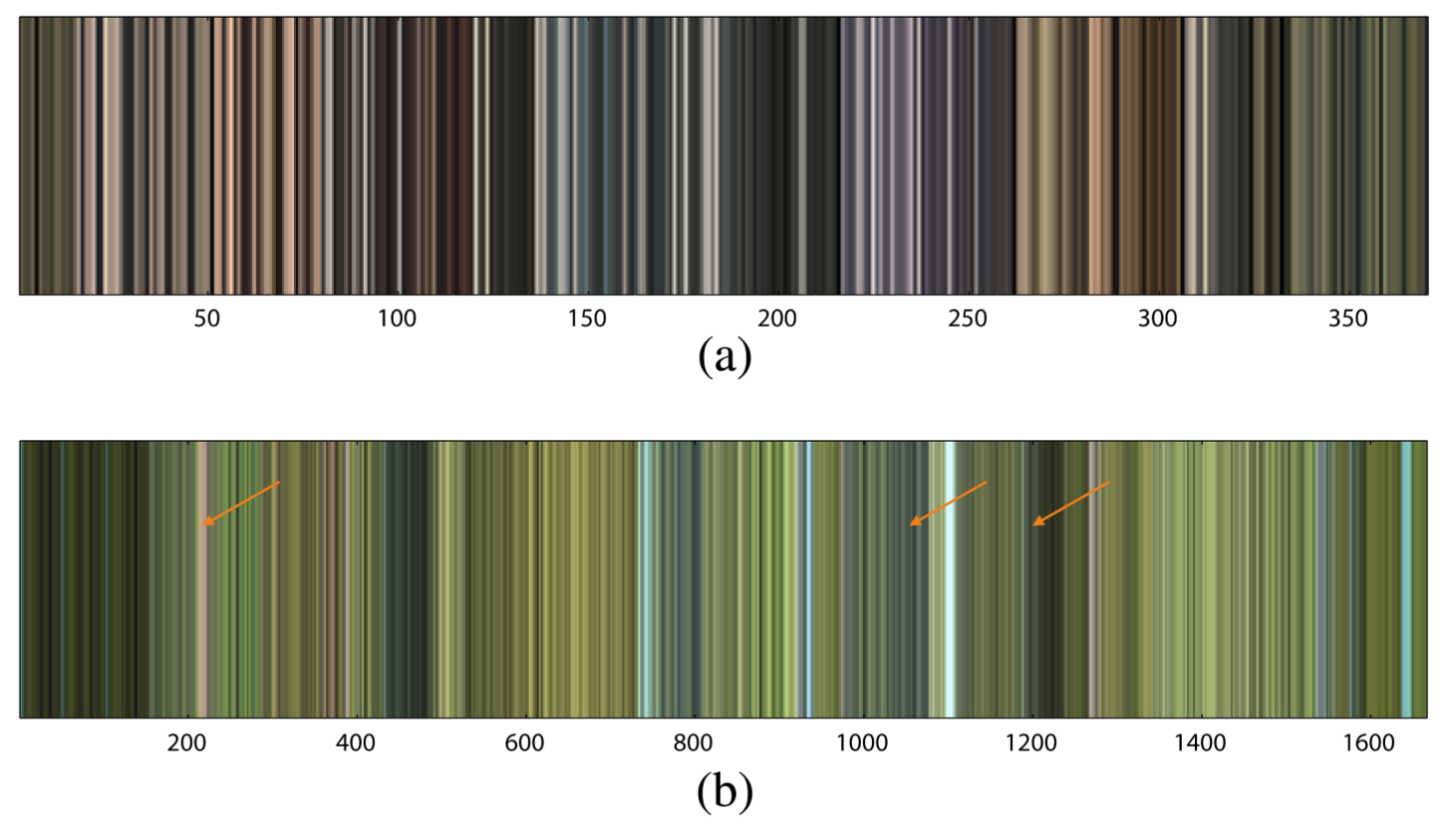
Foreground and background colors in the color model **(a)** Foreground colors representing elephant skin. **(b)** Background colors. Since the background has a larger diversity, the foreground colors are partly included in the set of background colors (arrows mark examples of such colors).

The resulting lists of foreground and background colors of all training images form the input to classifier training. Figure 5 shows the extracted foreground and background colors (in RGB for better visualization) over the entire training set. The list of background colors is larger than the list of foreground colors because the background consists of more segments. From Figure 5 we observe that the colors of the foreground are frequently contained also in the background (but more seldom vice versa) resulting in an asymmetry between both classes. The reason is that the background frequently contains colors similar to that of the elephants due to its large diversity (e.g., rocks, sandy grounds). From this observation it follows that color is a necessary but not a sufficient indicator for elephant detection.

We generate a discriminative color model by training a support vector machine (SVM) with a radial basis function (RBF) kernel from the foreground and background colors. Due to the asymmetry between the two sets of color, we assign the foreground class higher misclassification costs than the background class. This reduces the risk that the SVM misses a true elephant detection and at the same time, it increases the chance of false detections. The preferential treatment of the foreground class is intended at this stage of processing to keep the detection rate high. We handle false detections at a later stage of processing (see Section 3.6).

We observe that the RBF kernel separates both classes well. We set parameter gamma of the RBF kernel in a way that the number of support vectors is minimized. This assures a low complex decision boundary which increases the generalization ability of the classifier. The training error (estimated by fivefold cross-validation) is 92.83%. Experiments on test images show that the classifier detects segments that correspond to elephants with high accuracy. At the same time the number of false-positive detections is moderate. More results on the test data are presented in Section 5.1.

From the two sets of colors (see Figure 5), we observe that both sets occasionally contain very dark (near-black) and very bright (near-white) colors. For such colors a reasonable decision cannot be made by the classifier resulting in unreliable predictions. We apply a luminance filter to avoid these cases. Colors with near-black and near-white luminance are removed from the list of foreground colors. This assures that segments with colors near white or near black are rejected in elephant detection. We investigate the effect of luminance filtering in Section 5.4.

The color model presented in this section is completely adaptive to the provided training images. It does not make any assumptions about the underlying video material and is generally applicable to different objects of interest.

### 3.3 Color classification

The goal of color classification is to detect segments in the images of a sequence that are likely to belong to an elephant according to the trained color model. The emphasis of the color detector (as mentioned in Section 3.2) is primarily to maintain a high detection rate (no elephants should be missed), while a few false-positive detections are tolerated.

Each input image sequence is first preprocessed (resized and segmented) as described in Section 3.1. Next, we take the color (in LUV space) of each segment and classify the segments with the color model (without luminance filtering). We reject all segments that are predicted to belong to the background class and keep only segments predicted to be members of the foreground class. We refer to this approach as *one-stage classification* since classification is performed in one step.

**Figure 6:**
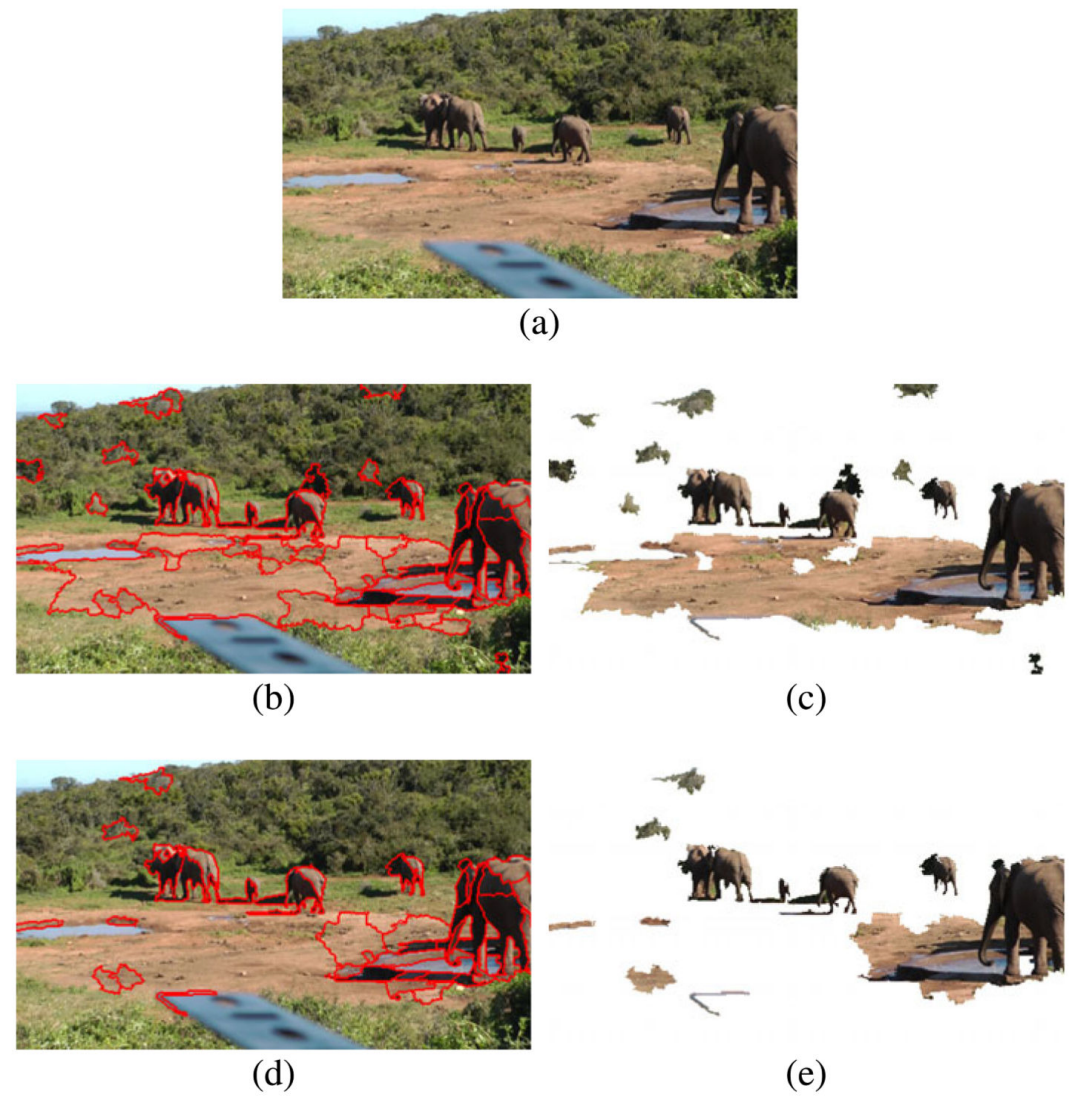
Color classification of an input image using two different classification schemes **(a)** Input image. **(b,c)** Results of one-stage classification. **(d,e)** Results of two-stage classification. Positively detected regions are highlighted by red contours in **(b)** and **(d)**. Panels **(c)** and **(e)** show the remaining segments in the image. The two-stage classification detects segments of elephants equally good as the one-stage approach but generates less false-positive detections.

**Figure 7:**
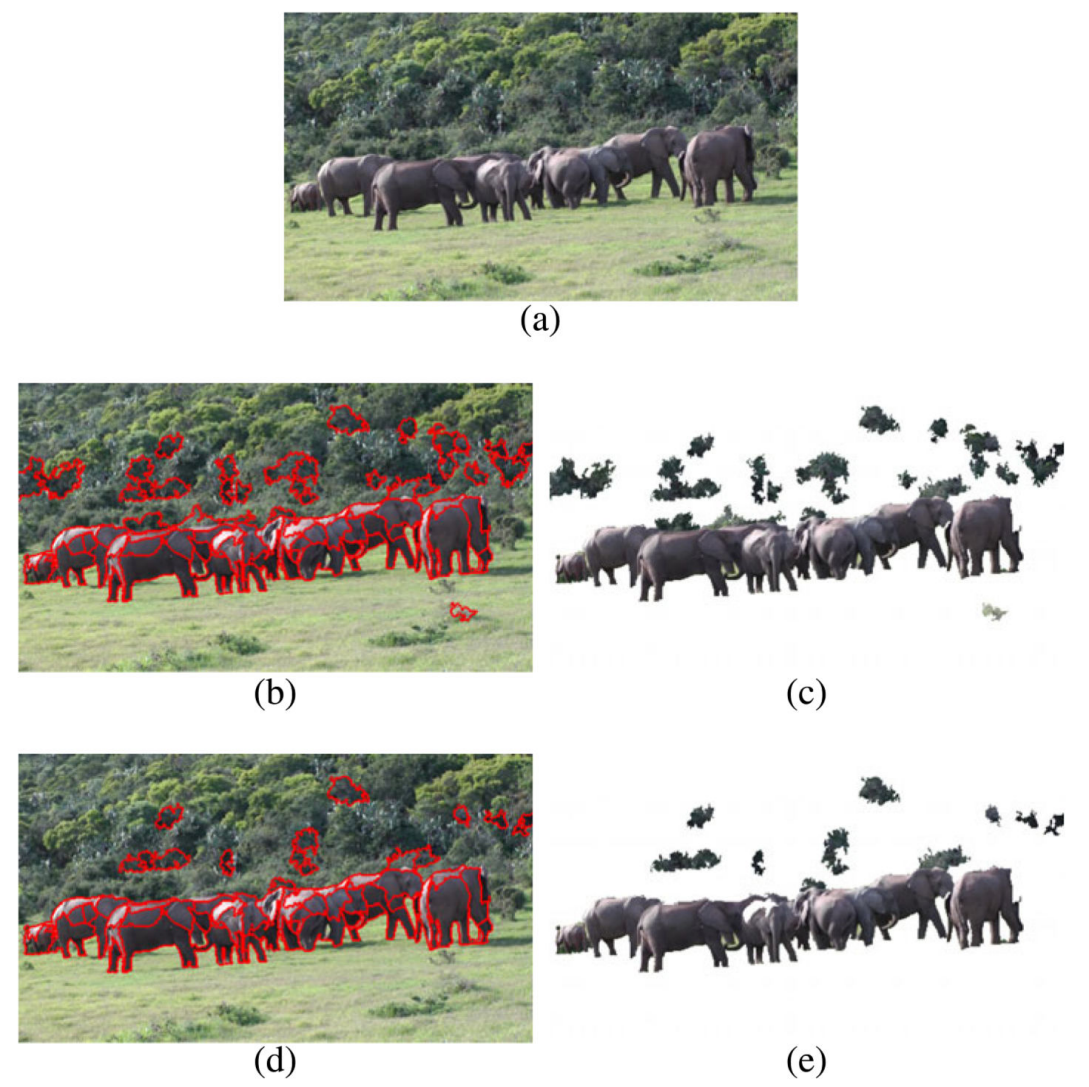
Color classification of an input image using two different classification schemes **(a)** Input image. **(b,c)** Results of one-stage classification. **(d,e)** Results of two-stage classification. Positively detected regions are highlighted by red contours in **(b)** and **(d)**. Panels **(c)** and **(e)** show the remaining segments in the image. Again two-stage classification is more robust than one-stage classification.

Results of color classification are shown in Figures 6b,c and 7b,c. From the detection results, we observe that the elephants are detected with high accuracy. At the same time many false-positive detections are generated, e.g., the ground in Figure 6c and the bushes in the background in Figure 7c. A closer look at the falsely detected segments reveals that their representative colors (mean color over the entire segment) resemble colors of elephants while the individual pixel colors have different characteristics. The mean color seems to be a suboptimal representation that removes too much information about the color distribution in the segments.

To compensate for this limitation, we propose a more fine-grained *two-stage classification* that operates on the individual pixels of a segment. First, we classify each pixel by the classifier used in one-stage classification. In a second step we apply a voting to the individual predictions. If the percentage of positively classified pixels is above two thirds, we classify the segment as positively detected; otherwise, we reject the entire segment. Results show that the two-stage classification is more robust in false detections while it detects elephants equally well. See Figures 6d,e and 7d,e for an illustration.

The result of color classification is a set of segments (candidate detections) that are likely to represent elephants in the scene. At this processing stage temporal relationships between the individual detections are not available. Another important clue for detection is temporal continuity. In the next step, we track the detected segments over time in order to temporally connect corresponding detections in different frames.

### 3.4 Tracking

**Figure 8:**
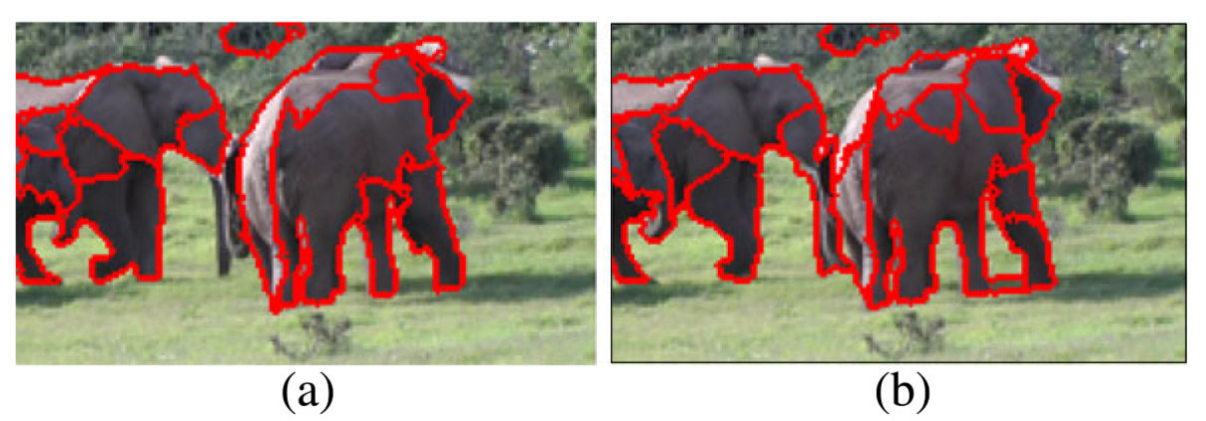
Segmentations of two consecutive frames **(a,b).** The segmentation in both frames is not consistent. Segments split and merge which can be observed from the highlighted contours.

The goal of tracking is to robustly detect elephants over longer time spans in a video sequence. For this purpose we have to establish temporal relationships between corresponding candidate detections in successive frames. The basis for tracking are the positively detected segments from Section 3.3. Tracking the segments is challenging since the frame-wise segmentation performed in preprocessing is temporally not always consistent. Reasons for temporal inconsistencies in the segmentations are differences in lighting, variations in the exposure of the camera, and object motions. Due to inconsistencies, a segment in one frame may be split into several segments in a following frame and vice versa (several segments in one frame may be merged in a following frame). Figure 8 shows inconsistencies in the segmentation of two successive frames. We propose a tracking scheme that handles these inconsistencies in a unified way. The proposed tracking scheme consists of four stages: (a) segment tracing, (b) trace intersection, (c) connectivity graph construction, and (d) subgraph extraction.

#### 3.4.1 Segment tracing

**Figure 9:**
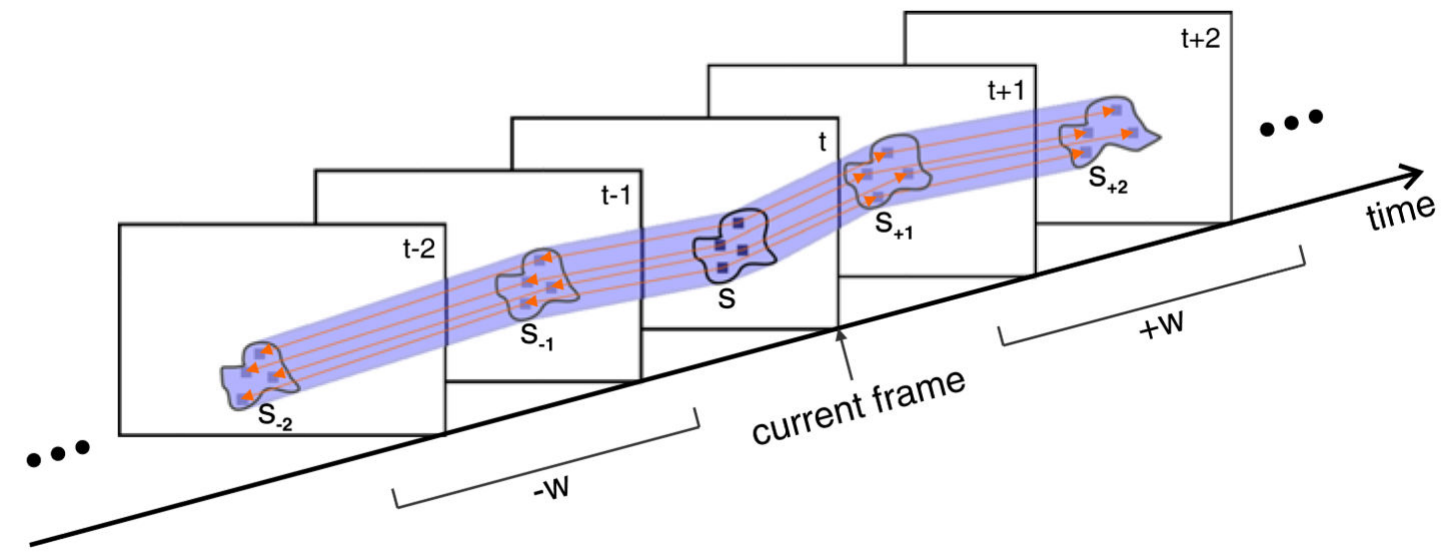
The process of region tracing A segment *s* is traced through a temporal window of ±*w* 2 frames by optical flow (orange arrows). The resulting trace (shaded violet area) of segment *s* contains the traced segments *s*_−2_, *s*_−1_, *s*_+1_, and *s*_+2_.

The first processing step consists of *tracing* a given input segment through the image sequence. Therefore, we solely rely on motion information and neglect the segmentations of the neighboring frames. The traced position and extent of a given segment in the next (or previous) frame is obtained by the optical flow of the segment’s pixels in the current frame [21]. We define a temporal analysis window of size *w* to limit the trace in time. Tracing is performed +*w* frames in forward direction and −*w* frames in backward direction. Tracing is iteratively performed from frame to frame. From tracing we obtain estimates of a segment’s position and extent in the surrounding frames. We call the set of all estimates the *trace* of the segment. Figure 9 illustrates the process of tracing. Tracing considers camera motion as well as deformations of the segments due to object motion. We apply tracing for all segments that are positively detected during color classification.

#### 3.4.2 Trace intersection

The traces are the basis for the establishment of temporal relationships between segments. For each frame in the temporal window of size *w*, we intersect the corresponding traced segment with the positively detected segments in the frame. For each segment we compute the area of intersection with the traced segment. The amount of intersection serves as a confidence measure for the establishment of temporal relationships. The confidence is computed as *c* = |*T* ∩ *S*| / |*T* ∪ *S*|, where *T* is the set containing all pixels covered by the traced segment and *S* is the set containing all pixels covered by the segment. The confidence corresponds to the portion of overlap between the trace and the segment. If the confidence between a segment and the trace is above a threshold *C*, we establish a temporal relationship (a link) between the intersecting segment and the *source* segment of the trace.

Tracking segments by the intersections of their traces has several advantages: (a) it implicitly handles cases where segments split and merge; (b) when temporal window sizes of *w >* 1 are used, temporal relationships over several frames (maximum *w*) can be established (in each direction). This enables the tracking of a segment even when it is missed for a few frames and then reappears; (c) from the temporal relationships established by trace intersection, we can derive spatial relationships between segments in the same frame (see Section 3.4.4).

**Figure 10:**
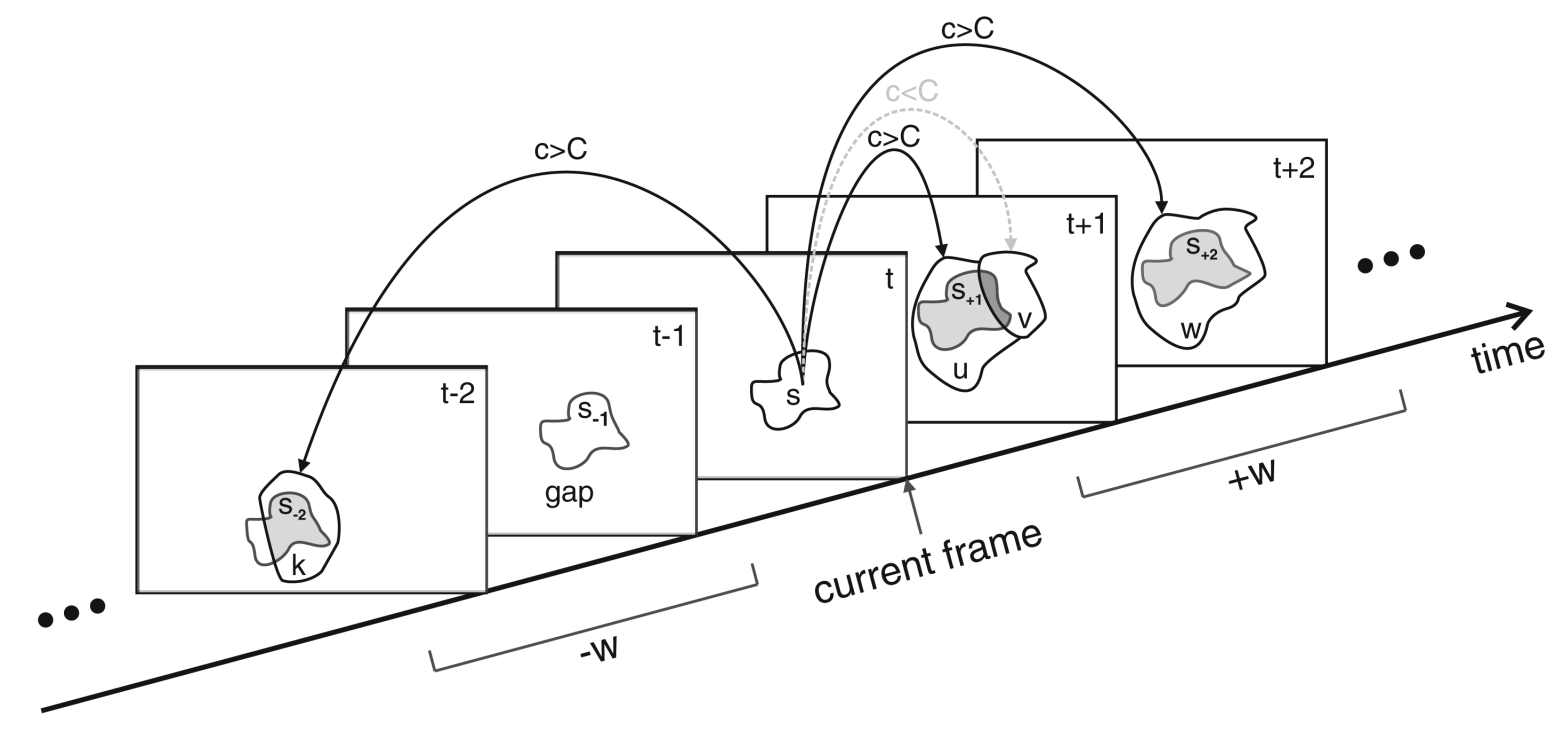
The process of trace intersection The trace of segment *s* is intersected with the segments k, u, v, and w in the neighboring frames. Gray regions represent the area of intersection with the trace. A temporal relationship (link) is established if the confidence *c* is higher than a threshold *C*. The trace intersection handles cases of splitting and merging as well as gaps in the temporal succession of segments.

Figure 10 illustrates the process of trace intersection. First, a segment *s* is traced through the temporal window (*w* = 2 in this example) resulting in a trace consisting of traced segments *s*_−2_, *s*_−1_, *s*_+1_, and *s*_+2_. For each frame of the temporal window, we intersect the traced segment with the positively detected segments in that frame. In frame *t* + 1, for example, the traced segment *s*_+1_ is intersected by two segments u and v. For both segments the confidence *c* of intersection is computed. Since the condition *c > C* is fulfilled for segment u, a link is established. For segment v the intersection is too small (*c < C*) and consequently no link is established. In frame *t* + 2 the segments u and v are merged into one segment w. Since the confidence of intersection is high, we establish a link from segment *s* to w. Links are also established in backward direction. For frame *t* − 1 no segment exists that intersects with the trace of segment *s* so no link can be established. However, in frame *t*−2 segment k intersects with the trace and a link is established between *s* and k. The link between *s* and k extends over the gap in frame *t* − 1. In this way the gap in frame *t* − 1 can be detected and handled correctly. We handle such gaps in tracking in a postprocessing step (see Section 3.7).

#### 3.4.3 Connectivity graph construction

Trace intersection is performed for all segments detected by color classification. The temporal relationships generated by trace intersection can be considered as a graph. Nodes in the graph are segments which are associated with a particular frame, and the edges in the graph are temporal relationships (links) between segments. The graph is directed since tracing generates forward- and backward-directed links. However, for the subsequent processing the direction of the edges is not important and thus we neglect their orientation. Due to splitting and merging of segments the graph may contain cycles. The density of the graph is dependent on the threshold *C* used in trace intersection (see Section 3.4.2). A higher (more stricter) threshold *C* impedes the creation of links and increases the sparsity of the graph, while a lower value of *C* facilitates the establishment of temporal relationships and increases the density of the graph.

**Figure 11:**
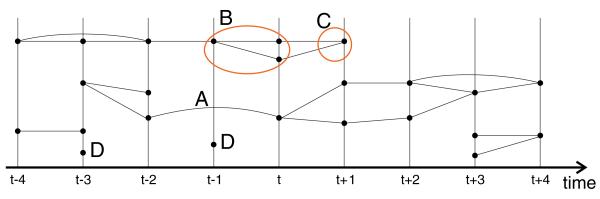
A connectivity graph constructed from temporal relationships Time progresses along the horizontal axis. Vertical lines represent frames and black dots are nodes of the graph (segments). Label A shows an edge that spans two frames, label B marks a case where segments are split. At the location of label C the segments merge again. Label D marks nodes that have no connected edges.

An example graph for a sequence of frames is illustrated in Figure 11. The graph can be arranged along the time axis since each node resides in a particular frame. The graph has edges that span one or more frames. Cycles indicate cases of splitting and merging. Two nodes in the graph have no connected edges. In practice such nodes correspond to unsteady segments with a short lifetime which are usually false detections from color classification.

#### 3.4.4 Subgraph extraction

The graph constructed in the previous section is sparse and consists of a number of disjoint subgraphs. The graph shown, for example, in Figure 11 consists of four disjoint subgraphs. Each subgraph represents the spatiotemporal track of a group of segments which are assumed to represent the same object.

We extract all subgraphs from the graph by a recursive procedure. For a given starting node (this can be an arbitrary node of the graph), we recursively traverse the entire graph and search for all nodes which are connected to this node. The resulting subgraph is removed from the original graph and the recursive search for the next subgraph in the remaining graph is performed. The procedure terminates when the remaining graph becomes empty.

**Figure 12:**
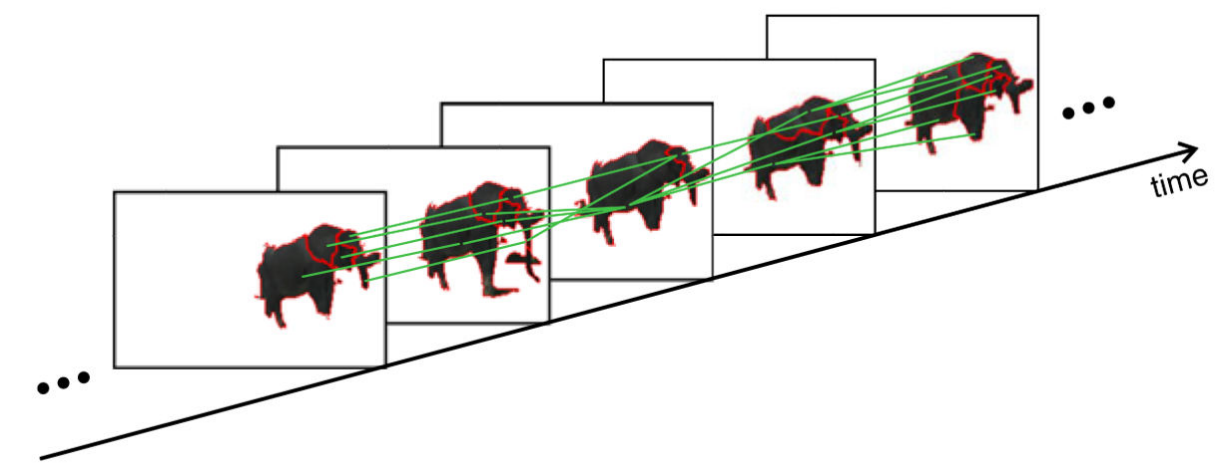
A subgraph extracted from a connectivity graph Individual segments have red contours. The green lines represent the edges (temporal relationships) in the subgraph. Temporal relationships that span several frames exist as well but are not shown to improve readability.

Figure 12 shows an example of a subgraph from one of our test sequences. The subgraph represents an individual elephant which is split into several segments. The segments are unsteady over time and frequently split and merge. The proposed tracking method (segment tracing and trace intersection) is able to compensate for the unsteady segmentation and correctly connects temporally coherent segments into one subgraph.

The subgraph provides useful information for detection and tracking. From the temporal relationships provided by the subgraph, we can infer *spatial* coherences between segments in the *same* frame. If for two segments from the same frame a connection exists somewhere in the subgraph (e.g., because the two segments are merged in a neighboring frame), this is a strong indicator that these two segments belong together and describe the same object.

**Figure 13:**
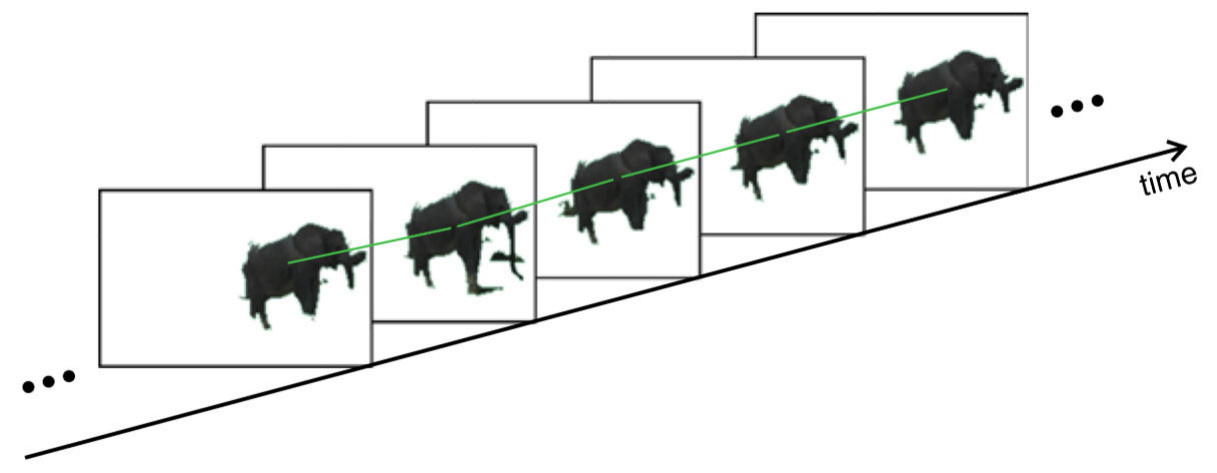
Spatial merging of a subgraph The resulting segments are more robust and more expressive than the original segments. All unnecessary edges in the graph are pruned. The result is a coherent spatiotemporal segment. The green lines represent the remaining edges (temporal relationships) in the subgraph.

We exploit the implicit spatial coherences in the subgraphs to refine the segmentation to obtain more robust detections. We spatially merge all segments in a frame which are connected by the same subgraph. The result is larger and more expressive segments representing the detected objects (see Figure 13). After merging, the subgraph represents a sequence of coherent segments. We regard such sequences in the following as *spatiotemporal segments*.

### 3.5 Spatiotemporal feature extraction

In Section 3.3 we point out that color is only a weak clue for the detection of elephants and that many false-positive detections are generated during color classification. The spatiotemporal segments obtained from tracking are spatially more meaningful than the original segments and additionally contain temporal information. They provide spatiotemporal clues which were not available during color classification and thus bear the potential to improve the quality of detection.

Each spatiotemporal segment represents a separate detection in the video sequence. The task is to decide whether a spatiotemporal segment is a false-positive detection or a true-positive detection. We extract spatiotemporal features from the segments to support this decision. We extract three different types of features: consistency, shape, and texture.

#### 3.5.1 Consistency features

Consistency features measure how long and how reliable a detection can be tracked. We extract two features: (a) the temporal *duration* (lifetime) of a spatiotemporal segment (the number of frames the segment can be tracked) and (b) the *instability* which is the portion of frames where a detection cannot be tracked during its lifetime (the portion of gaps that occur during tracking). The consistency features help to remove unreliable detections (with numerous gaps and short lifetimes) which often represent false positives.

#### 3.5.2 Shape features

The shape of elephants does usually not change abruptly. Slow changes in shape indicate correctly detected elephants while abrupt and fast changes rather suggest a false-positive detection. We design a feature that represents the variation of shape over time (*shape change*). First, we compute the area of a spatiotemporal segment at each frame which results in a series of areas *a* = *a*_1_, *a*_2_, *a*_3_, …, *a_n_*, where *n* is the number of frames spanned by a spatiotemporal segment. Next, we compute the difference between the maximum and the minimum of the areas and normalize this value by the maximum area: *f*_sc_ = *(*max*(a)* – min*(a))/* max*(a)*. The result is a value between 0 and 1, where 0 means that the area remains constant over time and higher values indicate strong temporal variations of the area.

#### 3.5.3 Texture features

The skin of elephants is poorly textured. Regions with strong texture and quickly changing texture are more likely to represent objects from the environment rather than elephant skin. We first compute the MPEG-7 edge histogram at each frame of a spatiotemporal segment. The edge histogram represents the distribution of differently oriented edges in a segment. It contains five bins: four bins for different orientations (horizontal, vertical, and two diagonal orientations) and an additional bin for nondirectional edges. For a spatiotemporal segment we obtain a series of edge histograms: *e* = *e*_1_, *e*_2_, *e*_3_, …*e_n_* where each histogram has five components, written as *e_i_*_,1_, *e_i_*_,2_, …, *e_i_*_,5_ for a histogram *e_i_* with 1 ≤ *i* ≤ *n*. Next, we derive two texture features from the edge histograms: (a) a measure for the edgeness [22] of a texture (*edge density*) and (b) a measure for the variation of texture over time (*texture variation*). The edge density *f*_ed_ is the mean of the summed histograms:

(1) $$f_{\mathrm{ed}}=\frac{1}{n}\sum_{i=1}^{n}\sum_{j=1}^{5}e_{i,j}.$$

The sum over an individual edge histogram corresponds to the portion of pixels in a segment that represent edges. Edge density represents the mean portion of pixels that represent edges over the entire spatiotemporal segment. The higher the edge density, the more textured is the corresponding spatiotemporal segment.

Texture variation *f*_tv_ is the mean over the value ranges of each individual histogram bin over time:

(2) $$f_{\mathrm{tv}}=\frac{1}{5}\sum_{j=1}^{5}\left(\max_{i=1}^{n}(e_{i,j})-\min_{i=1}^{n}(e_{i,j})\right).$$

First, the value range for each single bin of the histograms is computed. The mean over all bins provides an aggregated estimate of the temporal variation which is representative for the entire spatiotemporal segment.

### 3.6 Candidate validation

The goal of candidate validation is the improvement of detector robustness by the confirmation of correct detections and the rejection of false detections. This decision is based on the spatiotemporal features which allow for a temporal consistency analysis of the candidate detections. Note, that this consistency analysis does not require that the elephants actually move. The consistency analysis is applied to both, moving and static objects.

Each spatiotemporal segment represents one candidate detection. A spatiotemporal segment is either confirmed in its entirety or rejected in its entirety. Deciding over entire spatiotemporal segments exploits temporal information and thus is more robust than validating single (temporally disconnected) detections in a frame-wise manner. Candidate validation is based on the spatiotemporal features introduced in the previous section. First, individual decisions are made by thresholding each feature. Next, the individual decisions are combined into an overall decision for a candidate detection.

The determination of thresholds for automated analysis methods is a problematic issue for two reasons: First, thresholds increase the dependency on the input data and thus increase the risk of overfitting. Second, thresholds often depend on each other, e.g., when the decision by one threshold is the basis for a decision by a second threshold. Robust values for dependent thresholds cannot be determined separately from each other which in turn impedes model fitting and the evaluation of the method.

**Figure 14:**
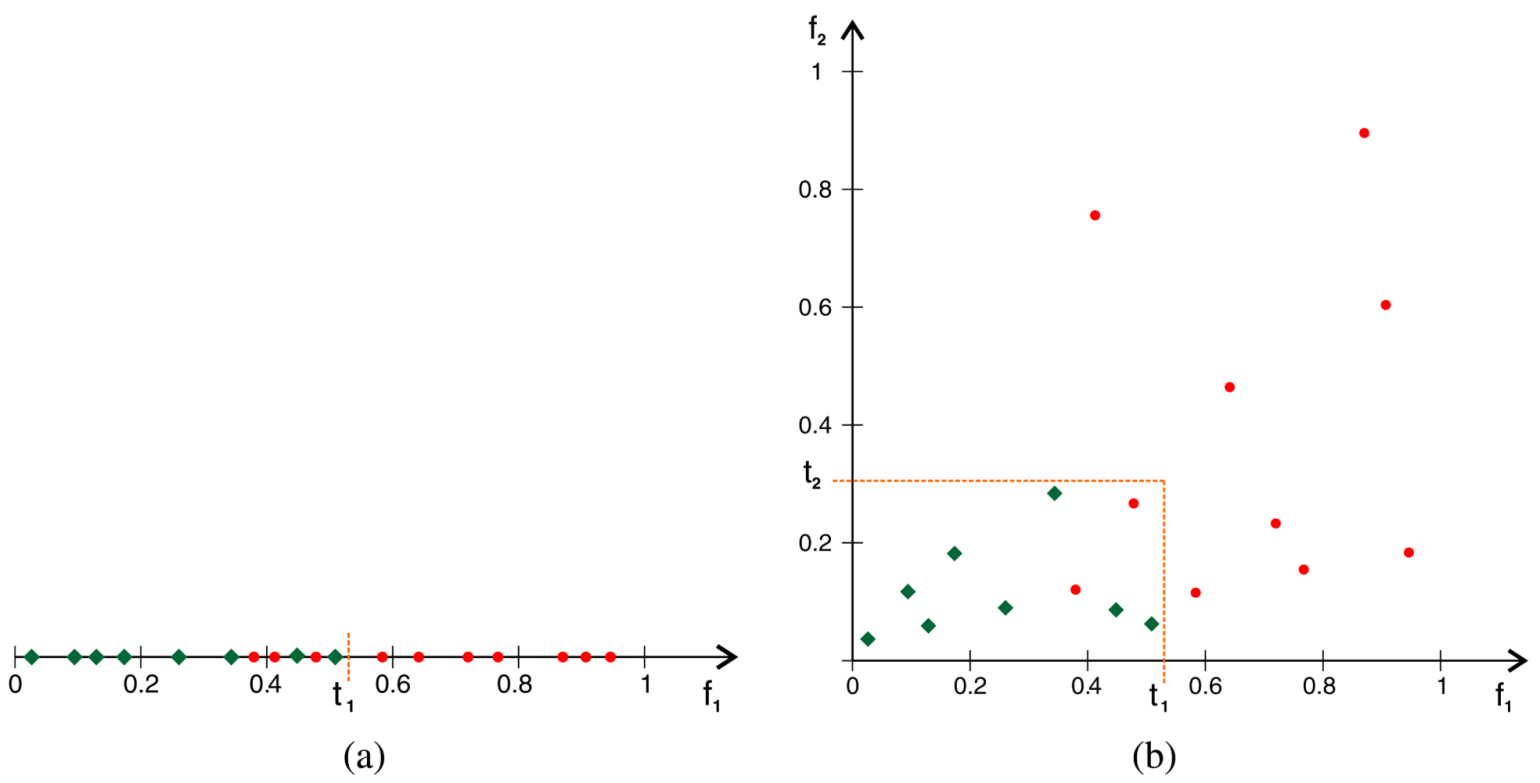
Threshold estimation and validation **(a)** The estimation of safe threshold value for a feature *f*_1_. True positives are represented as green diamonds, false positives are represented as red circles. The threshold *t*_1_ is increased (starting with 0) until all true positives are on one side (left) of the threshold. **(b)** The extension of **(a)** into two dimensions using an additional feature *f*_2_ with a corresponding threshold *t*_2_. Detections which are located in the rectangle bounded by both thresholds pass the validation (corresponding to a logical AND); all other detections are rejected.

The proposed validation scheme takes both issues into account. The thresholds for the features are determined independently from each other in a way that reduces the dependency on the data. Each threshold is set to a *safe* value that minimizes the risk of rejecting correct detections. This ‘safe’ value can be determined in a straightforward way: The threshold is initialized with the minimum value of the corresponding feature (lower limit of value range). Next, the threshold is increased subsequently by a constant step size. The threshold splits the value range of the feature into two subsets. The threshold value is fixed at *the* value which assures that (a) all true elephant detections remain in the same subset and (b) the size of this subset becomes minimal. Figure 14a illustrates the process of threshold estimation for a single feature. Each threshold value can easily be estimated in this way independently from the other thresholds using a few labeled ground-truth sequences.

For a given candidate detection, each feature is compared to its threshold. The resulting decisions are then combined using logical AND. This means that a spatiotemporal segment is confirmed as a positive detection if it passes all validations; otherwise, it is rejected. The logical AND combination assures that thresholds remain independent from each other and we do not have to investigate any interdependencies. The features capture different visual aspects (e.g., texture and shape) and thus complement each other for the rejection of false positives. The principle is illustrated in Figure 14. In Figure 14a three false detections (circles) pass the validation using *f*_1_ and threshold *t*_1_. Adding a second feature *f*_2_, as shown in Figure 14b, enables the correct rejection of an additional false detection due to the synergy of the two features.

The proposed validation scheme has several advantages: (a) each threshold value can be estimated separately, (b) the estimation of the thresholds using safe values is straightforward and reduces the dependency from the data, and (c) the logical AND combination of the single decisions exploits the complementary nature of the features.

In addition to the proposed validation scheme, we apply an SVM to the spatiotemporal features to reject false detections. The SVM is trained on a subset of video sequences using a cross-validation protocol. The trained classifier is then applied in the validation step instead of the proposed scheme. Since the required complexity of the decision boundary is not known during the design phase, we evaluate different kernels.

### 3.7 Postprocessing

**Figure 15:**
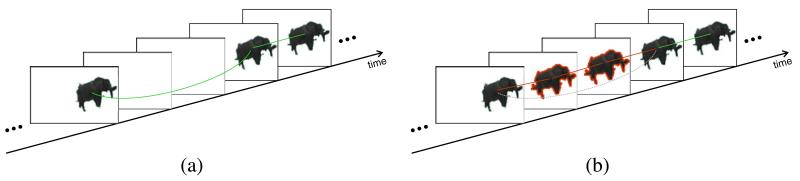
Closing gaps in tracking and interpolated segments Closing gaps in tracking for two frames where tracking was not successful **(a)**. Since tracking establishes temporal relationships over several frames, the gaps can be closed **(b)**. The segments (red contour) are interpolated using optical flow. The original link (dashed, gray) is replaced by new temporal links (solid, red). Green lines represent temporal relationships obtained by region tracking.

The detections that remain after candidate validation are considered to be positive detections of elephants. Due to noise, partial occlusions, and tracking failures, some detections cannot be tracked continuously over time and contain gaps (see Figure 15a). However, tracking proposed in Section 3.4 supports the establishment of temporal relationships over *several* frames. We exploit these long-time relationships to interpolate missing detections (see Figure 15b). For interpolation we employ the already available optical flow from segment tracing. New temporal relationships between the interpolated segments replace the original long-time relationships. Closing the gaps enables detection and tracking even if elephants are occluded for some time.

## 4 Experimental setup

In this section we introduce the video collection for the evaluation, the employed performance measures for quantitative evaluation, and the setup of the experiments.

### 4.1 Data

The analyzed data set is a corpus of videos captured by biologists during different field trips. The videos have been recorded during numerous field sessions in the *Addo Elephant National Park* (South Africa) in 2011 and 2012. During the recording sessions only handwritten field notes have been made which provide notes on selected events of interest and important observations. The generation of additional (more complete and systematic) descriptions during field sessions is out of scope due to temporal constraints. Consequently, the video data which are inputs to our method is temporally and spatially not indexed. It is unknown if and where elephants can be observed.

The videos are captured in high-definition format (1, 920 × 1, 080 pixels) at a rate of 25 frames per second. The entire data set contains about 150 GB of video files which corresponds to approximately 22 h of video and 2 million frames. For the evaluation of the approach we select a subset of the video collection. The main reason not to evaluate on the entire data set is that no ground truth is available for the data and the manual ground truth generation is extremely time-consuming.

We manually select a heterogeneous data set for evaluation that consists of 26 video sequences. The selected subset is representative for the data collection which in turn enables an objective evaluation of the approach. During selection we reject sequences which are too similar to the already selected ones to increase the heterogeneity in the data set. Figure 1 in Section 1 shows frames from selected sequences in the data set. The sequences contain elephants (groups and individual elephants) of different sizes (from far distance and intermediate distance to near distance). Elephants are visible in arbitrary poses and ages performing different activities, such as eating, drinking, running, and different bonding behaviors. The sequences show different locations, such as elephants at a water hole, elephants passing a trail, and highly occluded elephants in bushes. Sequences have been captured at different times of the day, in different lighting and weather conditions. Recording settings vary across the sequences from almost static camera (mounted on a tripod) to shaking handheld camera with pans and zooms. Additionally, there are sequences which contain no elephants at all and sequences where elephants enter and leave the scene.

**Figure 16:**
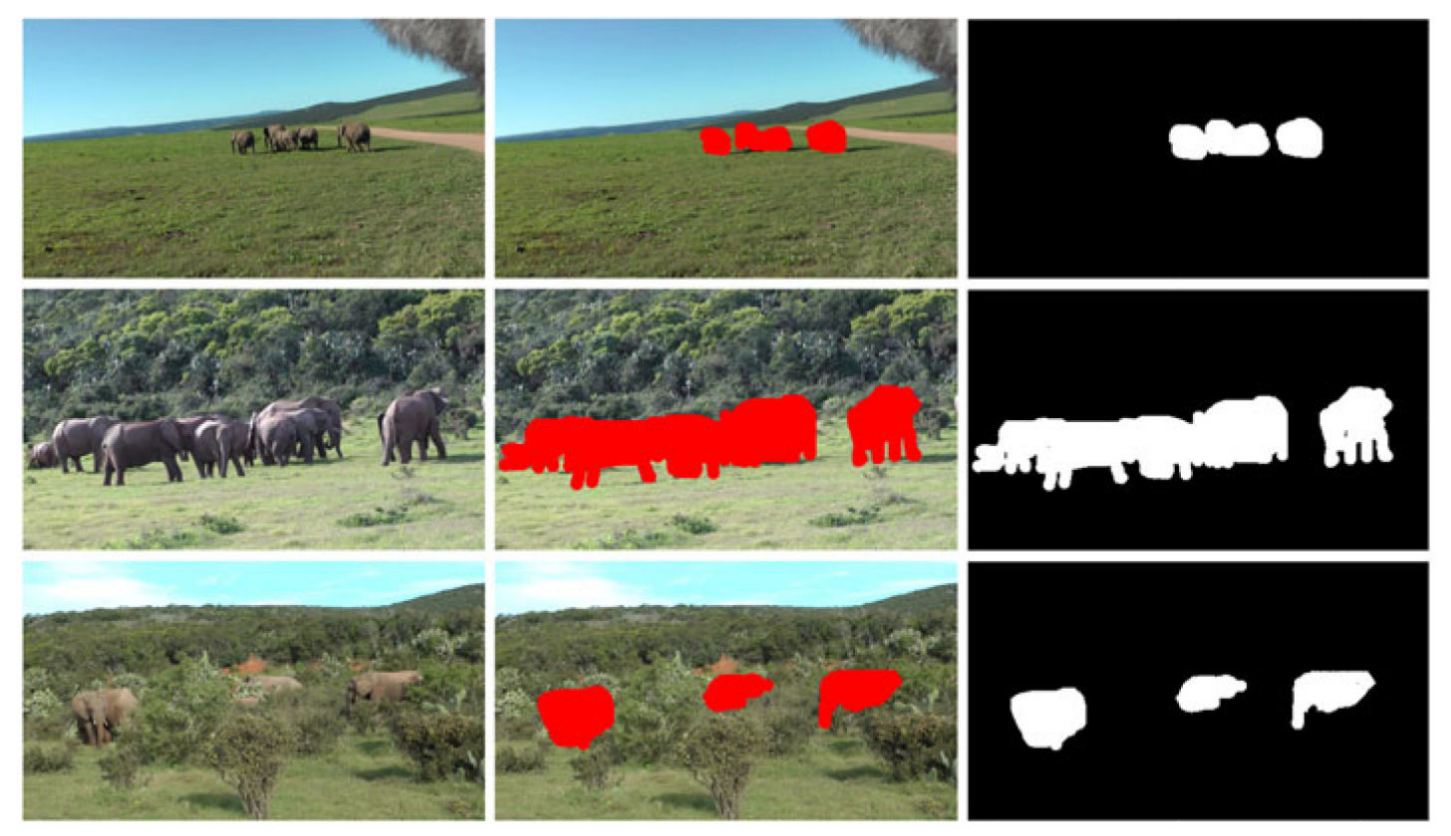
Ground truth for different sequences Left: original image, middle: labeled image, and right: ground truth mask.

For the quantitative evaluation of our approach, a ground truth of the data set is required. For elephant detection, a purely *temporal* ground truth, which provides only begin and end frame numbers of relevant time spans, would be sufficient in general. However, with a temporal ground truth, we are not able to evaluate whether or not a detection does actually spatially match an elephant. This impedes the objective computation of the false-positive detection rate. In order to compensate for this weakness, we create a spatiotemporal ground truth for each sequence in the data set. Each frame in a sequence is manually labeled and a ground truth mask is generated. Figure 16 shows ground truth masks for different sequences. The entire ground truth covers 715 frames and 1,751 manually labeled segments covering elephants. The ground truth contains moving elephants as well as resting elephants. Elephants whose image regions overlap are regarded as one ground truth segment. The accurate spatiotemporal ground truth enables the comprehensive evaluation of the detection performance of the approach.

The ground truth data are not only used for evaluation but also for training the color model introduced in Section 3.2. We exclude 16 randomly chosen images from the data set (this corresponds to 2% of the entire data set) and use them to train the color model. From the training images only individual pixel colors are used for training. Higher-level information such as spatial information is not used. This minimizes the dependency of the evaluation from the training data. Naturally, the training images for the color model are not used in the evaluation of the proposed method.

### 4.2 Evaluation measures

We evaluate the performance of the proposed approach for the *detection* of elephants. Note that this is different from evaluating the performance for the *segmentation* of elephants which is not the focus of our investigation. For elephant detection an elephant does not necessarily have to be segmented correctly to be successfully detected. We evaluate the detection performance spatially and temporally using the ground truth labels. We declare a detection to be successful if it coincides with a labeled ground truth region and thus with an image region covered by one or several (spatially overlapping) elephants. For performance estimation we compute the detection rate and the false-positive rate over the entire data set.

The detection rate is computed as the number of labeled ground truth (GT) regions hit by a detection divided by the total number of ground truth regions.

(3) $$\text{detection rate}=\frac{|\{\mathrm{GT}\;\text{regions hit}\}|}{|\{\mathrm{GT}\;\text{regions}\}|}.$$

Ground truth regions which are detected several times are counted only once. False detections are detections that contain background segments. The false-positive rate is the number of false detections divided by the total number of detections:

(4) $$\text{false positive rate}=\frac{|\{\text{false detections}\}|}{|\{\text{detections}\}|}.$$

### 4.3 Experiments

We systematically investigate the different components of the proposed approach. While we have presented intermediate qualitative results already in Section 3, a quantitative investigation of the components’ performance is necessary for an objective and comprehensive evaluation.

First, we investigate the performance of the approach using color classification only in Section 5.1. For this purpose we neglect temporal analysis and detect elephants using the color model introduced in Section 3.2. We investigate the discriminatory capabilities of the color model and compare the robustness of one-stage and two-stage classifications (see Section 3.3). For two-stage classification we further investigate the influence of different decision thresholds. The comparison of both classification schemes allows us to evaluate whether or not the additional processing costs of the two-stage classification are justified.

Second, we investigate how the robustness of the detector can be improved by temporal analysis in Section 5.2. We apply motion tracking and candidate validation by spatiotemporal features. We evaluate different combinations of spatiotemporal features to demonstrate the beneficial effect of their complementary nature. Additionally, we apply an SVM for candidate validation. The SVM is trained on the spatiotemporal features by using different kernels to discriminate positive and false-positive candidate detections. We perform cross-validation to evaluate the performance independently from the selection of the training data. Finally, we report the mean detection rate and mean false-positive rate over all cross-validation sets.

Third, we investigate the overall performance of the approach using different classifiers in Section 5.3. For this purpose, we apply SVMs with different kernels and compare the SVMs with nearest neighbor (NN), k-nearest neighbor (KNN), and linear discriminant analysis (LDA). We show that the ability of the classifiers to build robust color models varies significantly.

Fourth, the influence of different luminance filters (see Section 3.2) on the overall performance is evaluated in Section 5.4. We expect that luminance filtering improves the robustness of the approach, since it removes colors with particularly low and high luminance components which are often unreliably classified.

After systematic evaluation we present results for two different use cases which are provided by biologists. In both use cases automated elephant detection forms the basis for further investigations by the biologists. The first use case addresses the detection of elephants to assist biologists in detailed behavioral studies. Objects of interest in this use case are elephants at *intermediate* and *near* distance to the camera. Elephants far apart from the camera are not of interest since the individuals are too small for a detailed investigation of their behavior.

The second use case focuses on the detection of *distant* elephants in wide open areas. Biologists are interested in the presence of (groups of) elephants over wide surveyed areas. The detection of far-distant elephants should support biologists in the investigation of elephant groups, their sizes, and their migration routes. The objects of interest in this use case are significantly smaller than in the first use case which makes this task especially hard.

## 5 Results

We present evaluation results for different components of the proposed approach. Performance is measured in terms of detection rate (D) and false-positive rate (FP). We first present results for pure color classification. Next, we add temporal information and demonstrate the influence on performance. Additionally, we provide the overall performance using different classifiers and luminance filters. Finally, we present results for the investigated use cases (case 1 and case 2) presented in Section 4.3.

### 5.1 Pure color classification

Color classification is performed frame-by-frame and does not exploit temporal relationships between the segments. We compare the performance of one-stage classification and two-stage classification using the same classifier (an SVM with RBF kernel, trained as described in Section 3.2). Table 1 shows the results of color classification. For all evaluated configurations the detection rate is very high (between 94% and 97%). The SVM with RBF kernel is able to model the class of elephants well, although the positive training samples partly overlap with the samples of the background class (see Section 3.2). The false-positive rate shows larger variation across the different configurations than the detection rate. While one-stage classification yields a false-positive rate of 42%, in two-stage classification the false-positive rate drops by 10% to 32% at nearly the same detection rate. Two-stage classification clearly outperforms one-stage classification. This confirms our observations from Figures 6 and 7 in Section 3.3. We conclude that the additional computational effort of two-stage classification is justified.

**Figure 17:**
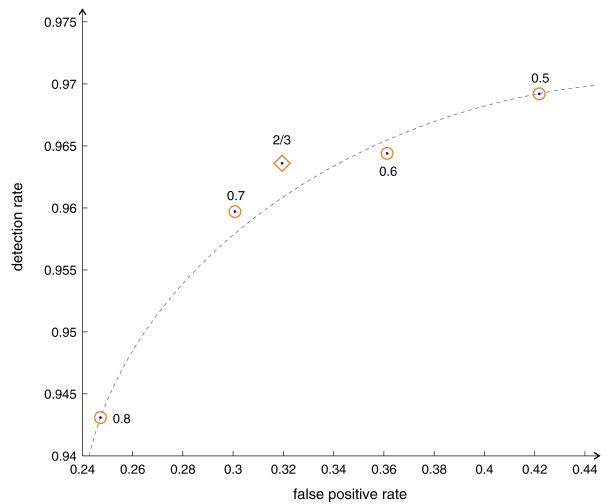
Performance of two-stage color classification for different decision thresholds The gray curve shows the relationship between false-positive rate and detection rate similar to a ROC curve. The graph shows that a decision threshold of two thirds (highlighted by a diamond) yields a robust trade-off between false-positive rate and detection rate compared to other thresholds (highlighted by circles).

In the two-stage classification, a segment is positively classified if more than two thirds of its area supports this decision. In the following, we investigate the robustness of this decision rule. We compute results for different decision thresholds (0.5, 0.6, 0.7, and 0.8) and estimate the robustness of detection. We plot the decision rate versus the false-positive rate (similar to a receiver operating characteristic curve) for all evaluated decision thresholds in Figure 17. The dashed line illustrates the (inverse) relationship between detection rate and false-positive rate. Good results in this graph are expected to be located in the upper left quarter of the graph. This is the case for the decision threshold of two thirds which yields a good trade-off between false-positive rate and detection rate.

From the results in Table 1, we observe that the false-positive rate is generally high. For the proposed two-stage classification, approximately each third detection is a false detection. This shows that color alone is a weak clue for elephant detection. Similar to [7] we observe that it is hard to build robust detectors from low-level information. An additional clue for detection is temporal continuity. In the following discussion, we investigate the potential of temporal analysis for automated detection.

### 5.2 Incorporation of temporal information

Temporal information is integrated into the detection process by the spatiotemporal features presented in Section 3.5. Table 2 shows results for different selections of spatiotemporal features. The first row shows the baseline without spatiotemporal features which is equivalent to two-stage color classification (see Table 1). First, we add the consistency features and observe a slight decrease of the false-positive rate by 2.7%. The consistency features remove unstable detections (noise) which cannot be tracked over larger time spans. Next, the shape feature (shape change) is added which reduces the false-positive rate significantly (by 7.9%). The shape feature removes detections in image regions where the underlying segmentation strongly varies over time which is, for example, the case in regions with smooth lighting transitions, such as sandy ground and trails. Finally, we add the texture features (texture variation and edge density) and observe a significant reduction of the false-positive rate by 14.8% to only 6.6% in total. The texture features remove false detections in highly textured background regions covered by bushes, trees, and plants.

The results in Table 2 show that the spatiotemporal features significantly increase the robustness of the detector. The false-positive rate is reduced in total by 25.4% to only 6.6%, while the detection rate remains relatively stable (96.4% versus 93.0%). Especially, the texture features are remarkable since they keep the detection rate constant and at the same time significantly reduce the false-positive rate. From Table 2 we further observe that the combination of different spatiotemporal features is highly beneficial for detector performance. The features are sensitive to different types of false detections since they represent complementary information.

We compare the proposed validation scheme with an alternative method based on an SVM as described in Section 4.3. We evaluate different kernels (linear, RBF, and polynomial) and optimize the respective hyperparameters using model selection. Additionally, we evaluate different subsets of the spatiotemporal features.

Results show that a linear SVM clearly outperforms the other kernels. The linear SVM yields a detection rate of 93% at a false-positive rate of 23%. The best result for the RBF kernel is obtained with a gamma of 1. The detection rate is 58% and the false-positive rate is 22%. The SVM with polynomial kernel performs suboptimal as well and yields a detection rate of 85% at a false-positive rate of 36%. In sum, the SVM-based method produces similar detection rates as the proposed validation scheme, however, the false-positive rate is significantly higher (22% versus 6.6%). Additionally, we evaluate different selections of spatiotemporal features. We observe that removing one or more features from the selection leads to a decreased performance. Optimal results are only obtained when all features are employed.

### 5.3 Robustness of classifiers

For color classification so far we have employed an SVM with RBF kernel. The RBF is able to model the complex decision boundary between the two input classes well and generates robust color models. The complex boundary is a result of the overlap between the two color classes mentioned in Section 3.2. We compare the performance to five alternative classifiers, namely, an SVM with a linear kernel, NN, KNN, LDA with a linear boundary, and LDA with a quadratic decision boundary. Table 3 provides the results for all classifiers. The overall performance of the linear SVM is below that of the SVM with RBF kernel. The linear SVM is not able to separate the overlapping distributions of the two classes. We observe less sparsity for the linear SVM, which means that more support vectors are required than for the RBF kernel. This indicates that the linear SVM is not able to model the decision boundary well and is prone to under-fitting. The RBF kernel provides more flexibility which allows for better modeling the boundary between the two classes.

The nearest neighbor classifier obtains a comparable detection rate as the SVM with RBF kernel. Unfortunately, the false-positive rate is higher (by 3.8%). Nevertheless, the result is impressive regarding the fact that the nearest neighbor classifier (in contrast to SVM) actually does not abstract from the training data. The KNN (with *K* = 5) performs similar to the linear SVM. The two versions of LDA perform suboptimally compared to SVM with RBF kernel. Similar to the linear SVM, the linear and quadratic boundaries of LDA are not able to model the complex boundary between the classes.

### 5.4 Filtering luminance

In Section 3.2 we point out that reasonable decisions cannot be made by the classifier for very dark and very bright colors. We propose to filter near-black and near-white colors during model generation to obtain more robust predictions. Table 4 provides detection results for different luminance filters. The first row shows the performance of the approach without luminance filtering (baseline). A medium luminance filter which removes samples below 10% of the minimum luminance and above 90% of the maximum luminance improves the false-positive rate by 1.4% (compared to the baseline) and keeps the detection rate constant. With an even stronger luminance filter we are able to halve the false-positive rate (from 5.2% to 2.5%) and still obtain a satisfactory detection rate of 91.7%. The luminance filter improves the robustness of the color model and thus positively influences the entire detection process.

### 5.5 Case 1: detection of elephants at intermediate and near distances

**Figure 18:**
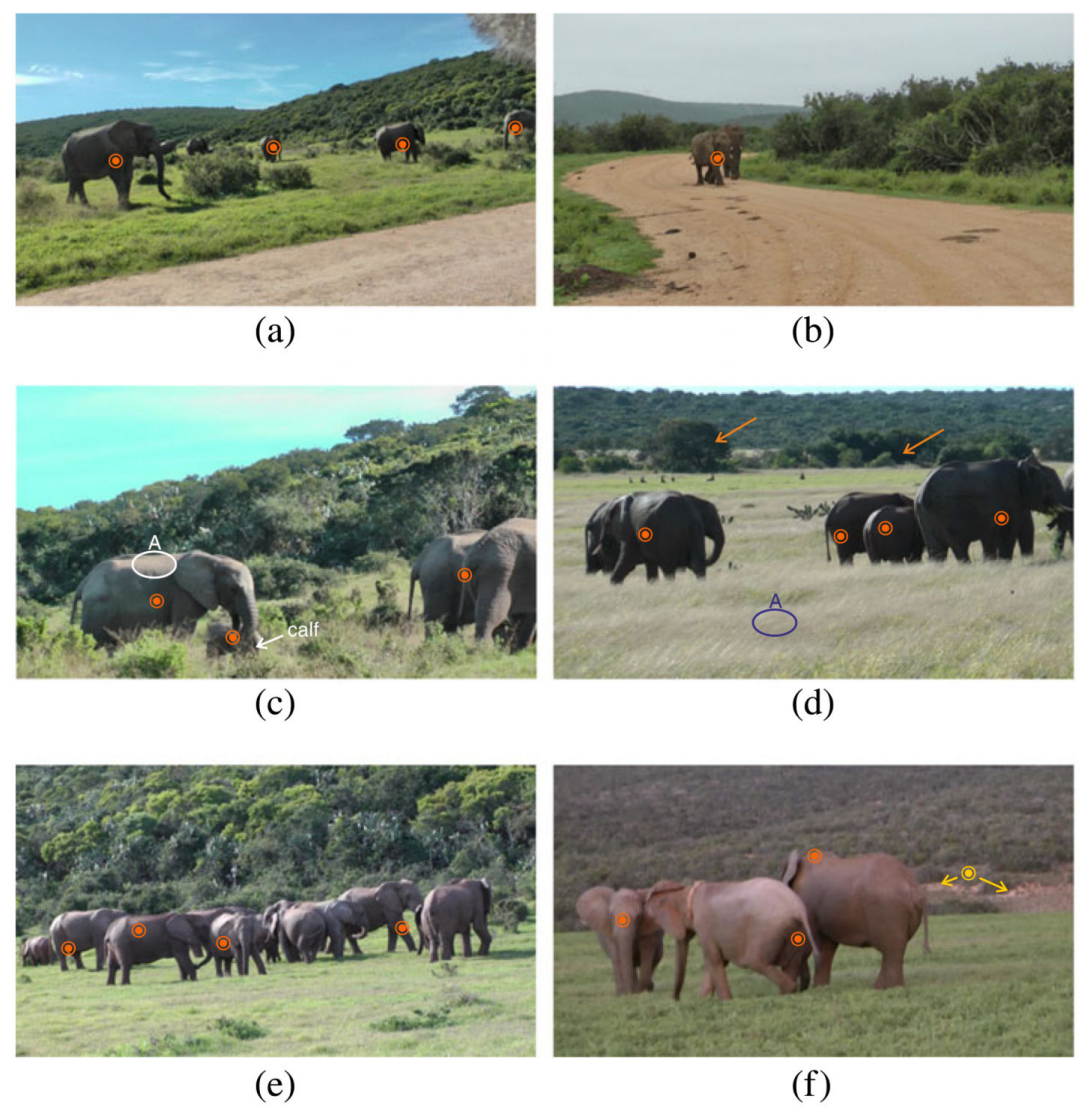
Results of elephant detection at intermediate and near distances to the camera True positive detections are labeled in orange and false-positive detections in yellow. **(a)** Group of elephants near a road. **(b)** Two elephants walking on a sandy trail. **(c,d)** Frames from sequences showing highly occluded individuals. **(e)** A group of elephants which is correctly detected several times. **(f)** A sequence with a false-detection (yellow) in the background that originates from a region that has a similar color to elephant skin.

The first investigated use case focuses on the detection of elephants at intermediate and near distances. For quantitative results, we refer the reader to the previous sections. In the following we present qualitative results and point out strengths and weaknesses of the approach. Figure 18a shows a group of elephants near a road. The sequence is hard for detection because the color of the road resembles the skin color of elephants in our color model. Additionally, the windshield of a microphone is visible in the top right corner. Both, the road and the windshield are predicted positively by color classification. However, we are able to remove these false detections during candidate validation since they have a significantly coarser texture than the elephants. Four out of five elephants can be detected successfully. One distant elephant is missed. While this elephant could be detected in some frames, the temporal coherence is too weak for a reliable detection.

Figure 18b shows two elephants walking on a sandy trail. The elephants do not set themselves apart from the background well (especially from the trail). While the color model produces false-positive detections on the trail, we are able to reject the entire trail during the candidate validation. The two elephants can be tracked reliably through the sequence. Note that we consider both elephants as one object to detect since they cover overlapping image regions.

Figure 18c,d shows frames from sequences where the elephants are relatively near to the camera. The proposed method robustly detects and tracks the animals over time. Figure 18c shows that the approach is able to detect elephants even if they are partly occluded: The calf in the lower left quarter of the image is widely occluded by grass and vegetation but can be detected and tracked successfully. Figure 18d shows a backlight scene where the elephant skin exhibits particularly low contrast. While the elephants are detected remarkably well, no false detections are made in similar dark background regions (labeled by arrows). The grass in the foreground of Figure 18d is likely to produce false detections because the color resembles that of sunlit elephant skin. This becomes evident when we compare the colors surrounding label ‘A’ in Figure 18c,d. False detections in such areas cannot be distinguished by color. However, additional texture and shape clues enable the rejection of false positives in this area.

Figure 18e shows a group of elephants which has previously been shown in Figure 7 in Section 3.3. From Figure 7d,e we observe numerous false detections of color classification in the background. Figure 18e shows the result after temporal analysis. The false detections in the background are temporally not stable and are removed by consistency constraints, while the detection of the elephant group remains stable.

Figure 18f shows an image with a false detection in the background (yellow). The false detection originates from the earthy area around the detection (labeled with arrows) and cannot be removed during candidate validation. The three elephants in the sequence are tracked consistently through the sequence.

**Figure 19:**
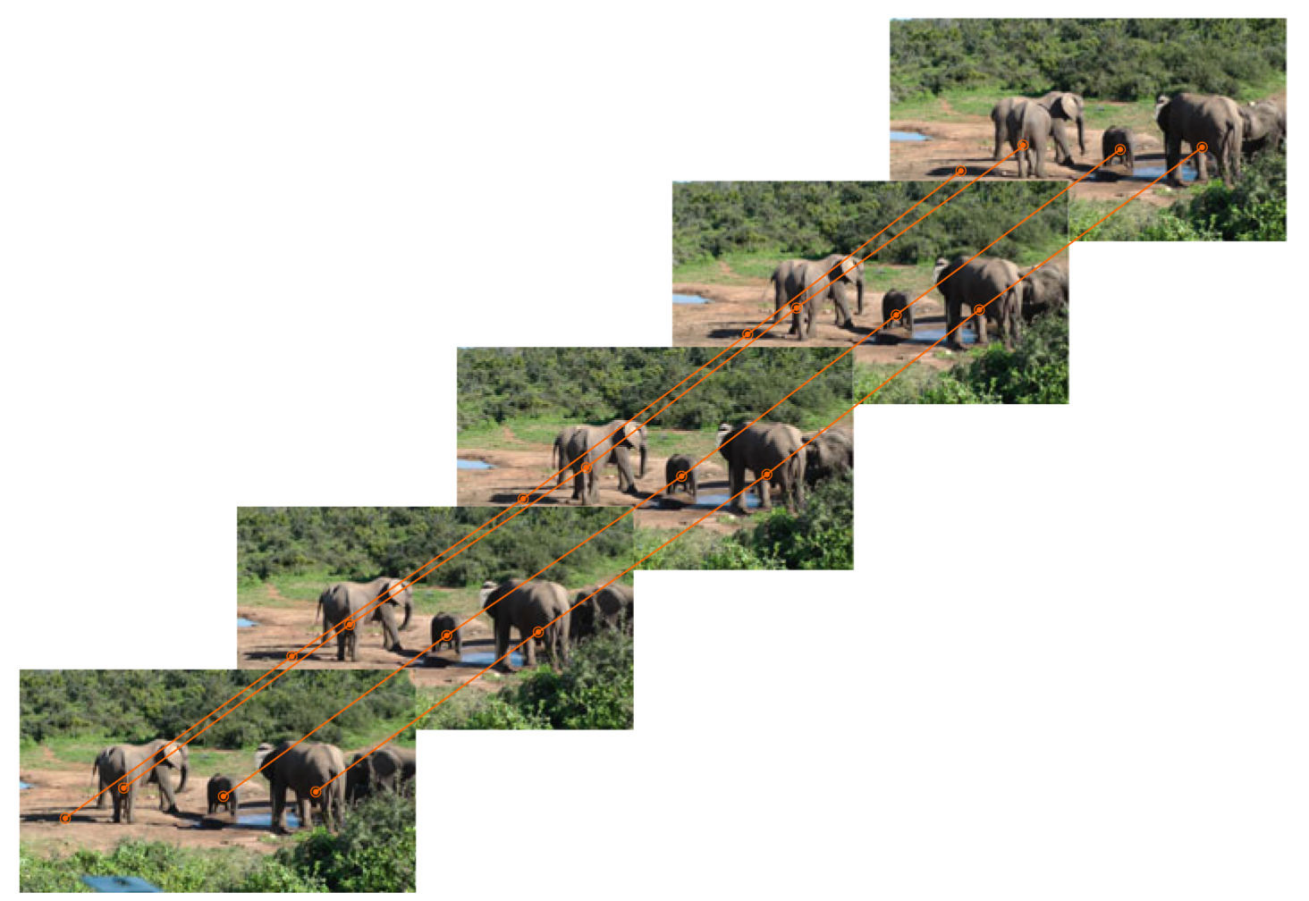
Tracking elephants through an image sequence The orange lines connect the matched detections across two frames. Note the camera pan to the left which is best recognized from the horizontal shift of the background. We skip intermediate frames for better visibility.

From motion analysis we obtain all information necessary to track elephants over time. For most sequences tracking is successful. Potential tracking failures are eliminated by interpolation during postprocessing (Section 3.7). Figure 19 shows tracking results for a sequence showing a group of elephants at a water hole which has been shot by a handheld camera. While the camera slowly pans to the left, the elephants are tracked consistently.

### 5.6 Case 2: detection of elephants at far distances

The detection of far-distant elephants is challenging for two reasons. First, due to the small size of the elephants, the image must be segmented at a finer scale during preprocessing to assure that each elephant is represented by at least one segment. However, due to this fine-grained segmentation, the number of segments grows significantly. At the same time the portion of segments related to elephants decreases drastically due to the small size of the elephants. Thus, detecting elephants becomes significantly harder and the probability of false detections increases. Second, small image segments are less expressive and exhibit less distinctive features then larger segments which impedes the automated detection.

For the detection of distant elephants, we decrease the minimum size of an image segment to 20 pixels during segmentation. Note that for experiments on intermediate and near-distant elephants, a minimum size of 150 pixels is adequate (at the employed video resolution). In quantitative experiments we obtain a detection rate of 88% at a false-positive rate of 39%. While the detection rate is satisfactory, the false-positive rate is high compared to previous experiments. This is a result of the finer segmentation which significantly increases the complexity of the task.

**Figure 20:**
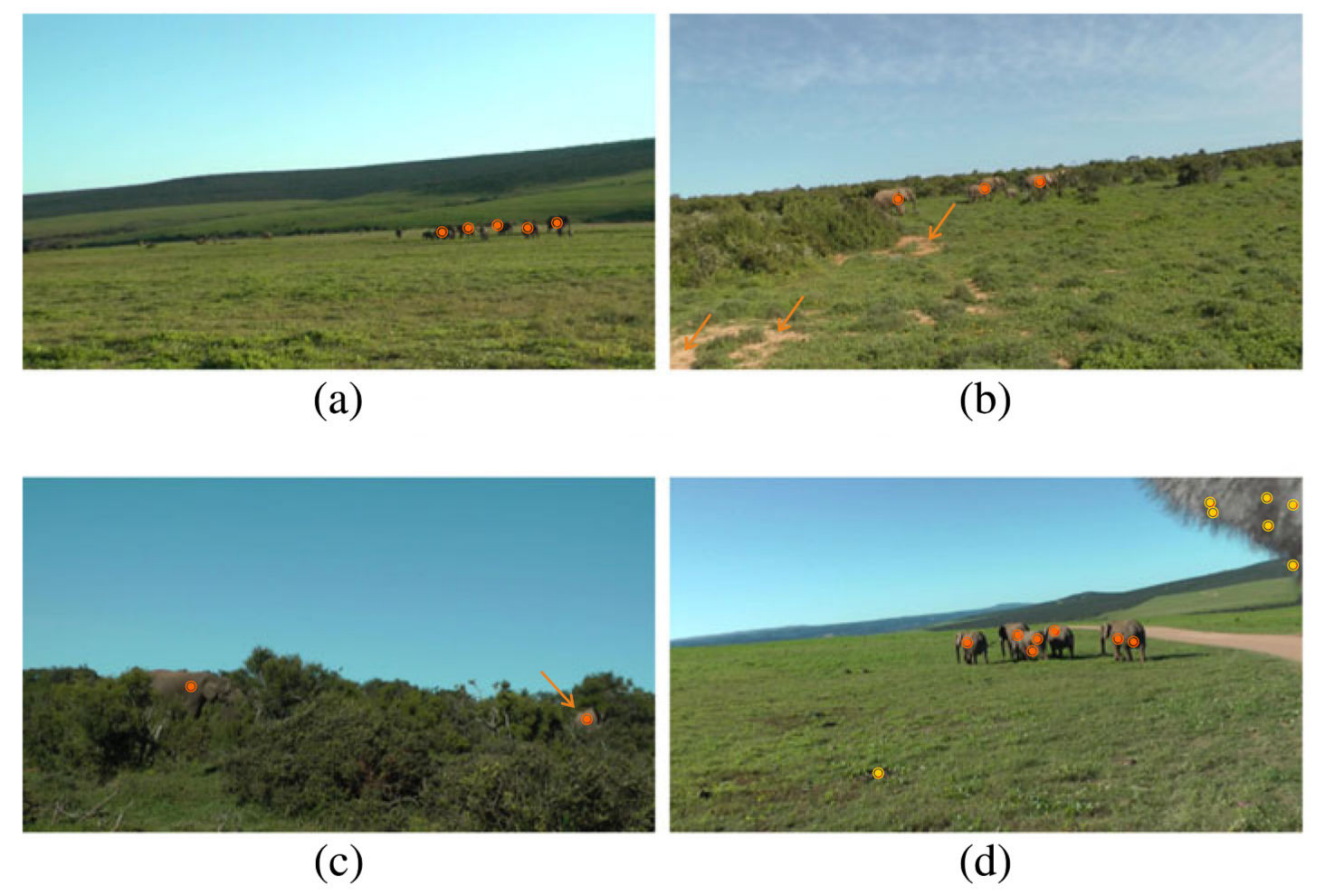
Results for the detection of elephants which are far apart from the camera True positive detections are labeled in orange and false-positive detections in yellow. **(a)** A group of seven far-distant elephants. **(b)** Small group of partly occluded elephants in the field. **(c)** Highly occluded elephants. **(d)** False detections in an image region which has a high similarity to elephant skin color.

Results for different sequences contained in the quantitative evaluation are shown in Figure 20. Figure 20a shows a group of seven elephants. Five of the elephants can be detected and tracked successfully. Two elephants at the left side of the group are too small for detection (at least at the employed video resolution).

Figure 20b shows a small group of elephants in the field. All regions covered by elephants are detected correctly. Note that no false positives are generated in regions (labeled with arrows) which have nearly the same color as the elephants.

In Figure 20c, a scene with large occlusions is shown. The depicted scene demonstrates well that shape is not a valid cue for the detection of elephants. Although the elephants are mostly occluded (especially the right one, labeled with an arrow), we are able to robustly detect and track them through the sequence.

An example where the detector generates inaccurate results is provided in Figure 20d. Additionally, to correct elephant detections, a number of false-positive detections are returned. One false-positive detection is located in a darker area in the grassland. The other false positives are located in the upper right corner which is covered by the windshield of a microphone that extends into the view of the camera. In the detection process the fine-grained segmentation splits the area covered by the windshield into numerous small segments. The small segments exhibit only weak texture clues. As a consequence, they are not rejected during candidate validation.

The presented results demonstrate both the capabilities and the limitations of the proposed approach. We are able to robustly detect elephants with high accuracy, which shows that the approach is well-suited to support biologists in their investigations. We yield a low false-positive rate for the detection of elephants at intermediate and near distances. The detection of far-distant elephants demonstrates the limitations of the approach. Due to the fine granularity of the analysis, the number of false positives increases. However, most false positives are reasonable and occur in areas where they would be expected. Aside from false positives, we are able to detect and track most elephants even if they are occluded or represented only by a small image area.

## 6 Conclusions

The contribution of this work is a reliable method for the detection and tracking of elephants applicable to unconstrained wildlife video. Unlike related approaches, we do not make strong assumptions about the video material and the environment, such as the number of animals present, their poses to the camera, the amount of background clutter, and the camera operation. As a consequence, we are able to detect and track elephants of different sizes and poses in their natural habitat. The approach robustly handles occlusions and detects elephants even if most of their bodies are hidden, e.g., behind vegetation. Experiments show that robust and accurate detection is possible in heterogeneous scenarios at a remarkable small false-positive rate of only 2.5%. We reach the limits of the approach by the detection of far-distant elephants. While the detection rate in this case is still high, the sensitivity to false positives grows. We conclude that this use case requires the integration of additional constraints related to the shape and size of far-distant elephants.

The major benefit of this work is a novel approach that enables the automated indexing of unconstrained wildlife video. As an additional information, our approach provides the spatial location and complete tracking information for each detection. This makes the approach a sound basis for higher-level analysis tasks, from the automated estimation of group sizes, to the identification of animals, and to the automated recognition of different activities and behaviors.

## Figures and Tables

**Table 1:** Performance of different color classification schemes (one-stage classification vs. two-stage classification with different decision rules)

Scheme	Rule	D(%)	FP(%)
One-stage	-	96.9	42.0
Two-stage	2/3	96.4	32.0
Two-stage	0.5	96.9	42.2
Two-stage	0.6	96.4	36.1
Two-stage	0.7	96.0	30.1
Two-stage	0.8	94.3	24.7

**Table 2:** The effect of temporal information on the overall performance

Spatiotemporal features	D (%)	FP (%)
None	96.4	32.0
Consistency	94.7	29.3
Consistency + shape	93.0	21.4
Consistency + shape + texture	93.0	6.6

The combination of different spatiotemporal features improves results significantly which shows that the features complement each other.

**Table 3:** The effect of different classifiers on detection performance

Classifier	D(%)	FP (%)
SVM RBF	93.0	6.6
Linear SVM	82.7	5.5
NN	93.4	10.4
KNN	84.9	5.6
LDA (linear)	91.2	10.6
LDA (quadratic)	88.9	13.9

The SVM with RBF kernel outperforms all other evaluated classifiers.

**Table 4:** The potential of luminance filtering

Luminance filter	Lower limit(%)	Upper limit(%)	D(%)	FP(%)
Off	0	100	93.0	6.6
Medium	10	90	93.0	5.2
Strong	20	80	91.7	2.5

Luminance filtering reduces the number of false detections and thus improves the robustness of detection.
